# The Effect of Increasing Inclusion Levels of a Fucoidan-Rich Extract Derived from *Ascophyllum nodosum* on Growth Performance and Aspects of Intestinal Health of Pigs Post-Weaning

**DOI:** 10.3390/md17120680

**Published:** 2019-11-30

**Authors:** Ruth Rattigan, Torres Sweeney, Stafford Vigors, Kevin Thornton, Gaurav Rajauria, John V O’Doherty

**Affiliations:** 1 School of Agriculture and Food Science, University College Dublin, Belfield, 4 Dublin, Ireland; ruth.rattigan@ucdconnect.ie (R.R.); staffordvigors1@ucd.ie (S.V.); gaurav.rajauria@ucd.ie (G.R.); 2 School of Veterinary Medicine, University College Dublin, Belfield, 4 Dublin, Ireland; torres.sweeney@ucd.ie (T.S.); kevin.thornton@ucd.ie (K.T.)

**Keywords:** pigs, post-weaning diarrhoea, fucoidan, microbiota, growth performance

## Abstract

This study examines the effects of increasing dietary inclusion levels of fucoidan, from a 44% fucoidan extract on the growth performance and intestinal health of pigs post-weaning (PW). Seventy-two newly weaned pigs (8.4 kg (SD 1.06)) were assigned to: (T1) basal diet (BD); (T2) BD + 125 ppm fucoidan; (T3) BD + 250 ppm fucoidan (8 pens/treatment). The appropriate quantity of a 44% fucoidan extract was included to achieve these inclusion levels. Faecal scores were recorded daily. On d15 PW, samples were collected from the intestinal tract from 1 pig/pen from the BD and BD + 250 ppm fucoidan groups. Pigs supplemented with 250 ppm fucoidan had improved faecal scores and increased concentrations of total volatile fatty acids and propionate in the colon (*p* < 0.05). The fucoidan-rich extract reduced the expression of *CLDN5* (duodenum), *SCL5A1*/*SGLT1* and *SI* (jejunum) and *TJP1*, *FABP2*, and *SLC5A1* (ileum) (*p* < 0.05). The extract reduced the relative abundance of *Prevotella* and *Lachnospiraceae* (*p* < 0.05) and increased the abundance of *Helicobacter* (*p* < 0.01) in the caecum. However, no negative impact on growth performance or small intestinal morphology was observed. Thus, the inclusion of 250 ppm fucoidan improves faecal consistency without affecting growth performance and therefore warrants further investigation as a supplement for the prevention of PW diarrhoea under more challenging commercial conditions.

## 1. Introduction

On modern commercial pig farms, weaning is an abrupt process, resulting in severe stress and a transient reduction in feed intake and growth rates [1,2,3]. This stress increases the piglet’s susceptibility to gastrointestinal disturbance, which is characterised by inflammation, increased epithelial permeability, and the maldigestion and malabsorption of nutrients. The proliferation of pathogenic bacteria in particular enterotoxigenic *Escherichia coli* [3,4] often leads to post-weaning diarrhoea. Traditionally, antibiotic growth promoters (AGP) and pharmacological doses of zinc oxide (ZnO) have been used to supplement the diets of newly weaned pigs to enhance growth and prevent the proliferation of pathogenic bacteria. However, owing to the role of AGP in the rise of antimicrobial resistance, the EU banned the use of AGPs in 2006 (EC Regulation no. 1831/2003). Now concerns surrounding the relationship between ZnO and antimicrobial resistance and the risk of environmental accumulation have led to a decision to ban pharmacological doses of ZnO in the EU from 2022 (Commission Implementing Decision of 26.6.2017, C (2017) 4529 Final). In addition, the EU will also implement further restrictions on the use of antibiotics for the treatment of groups of animals from 2022 [5]. Thus, identifying natural alternatives will be important if we are to maintain future animal growth and health during the turbulent post-weaning period. 

Brown seaweeds such as *Ascophyllum nodosum* are a valuable source of bioactive polysaccharides, including laminarin, alginates, mannitol, phlorotannins and fucoidans [6]. Fucoidans are sulphated, fucose rich polymers present in the algal cell wall [7]. They are chemically complex polysaccharides with varying compositions and molecular weights, but typically comprise a backbone of (1→3)-linked α-l-fucopyranosyl or of alternating (1→3)- and (1→4)-linked α-l-fucopyranosyl residues [8]. Identified biological activities include antimicrobial, immunomodulatory, antioxidant and antiviral effects [9,10,11], and as fucoidan is a non-digestible polysaccharide, it may also have prebiotic effects [6]. In pigs, fucoidan increased lactobacilli in the caecal digesta [12], colonic digesta [13] and faeces [14,15] and caecal and colonic butyrate concentrations [12]. The gastrointestinal microbiota contributes to host health and growth through the fermentation of carbohydrates, production of vitamins, healthy maintenance of the intestinal epithelium, immune and neural system development in neonates and protection from opportunistic pathogenic bacteria [16,17]. Thus, dietary supplementation with fucoidan may beneficially influence the composition of the intestinal microbiota of the newly weaned pig and thus, prevent the overgrowth of pathogenic bacteria and the onset of post-weaning diarrhoea.

However, the polysaccharide content and composition of seaweeds vary with season, the region of harvest, macroalgal species and extraction procedure [18,19]. Ascophyllum nodosum has been reported to contain 4%–11% fucoidan, 24%–28% alginate, 5%–11% mannitol and 1%–10% laminarin [6]. While research is under way to develop new innovative extraction methodologies to achieve high yields of purified polysaccharides, the currently used traditional extraction and purification processes are costly and inefficient in terms of energy usage and time (as reviewed by [20]). Thus, the first objective of this study is to determine the effect of increasing fucoidan inclusion levels from an extract containing 44% fucoidan from *Ascophyllum nodosum* on the faecal scores and large intestinal microbiota of weaned pigs. It is hypothesised that the optimum inclusion level of fucoidan will enhance the large intestinal microbiota, thereby reducing the incidence of post-weaning diarrhoea. Previously increasing dietary inclusion of an *Ascophyllum nodosum* extract led to a linear reduction in the daily gains of grower finisher pigs associated with reduced diet digestibility [21]. As the digestive system of the pig is not fully developed at weaning, higher inclusion levels may negatively impact pig growth performance and small intestinal functionality due to the increased levels of non-digestible fibre present (fucoidan (44%) and alginates (13.5%)). Thus, a further objective of this study is to identify the effects of the fucoidan-rich extract on growth performance and parameters related to small intestinal health including morphology, the expression of genes involved in nutrient digestion and absorption, inflammation, mucus production, pathogen recognition and tight junctions.

## 2. Results

### 2.1. Performance and Faecal Scores

This study investigated the effects of increasing dietary inclusion levels of a fucoidan-rich extract containing 44% fucoidan, 2.59% laminarin, 13.5% alginates, 4.38% mannitol, 3.48% phlorotannins and 31.95% ash on pig growth performance and faecal scores in the first 14 days post-weaning. The effects on average daily gain (ADG), average daily feed intake (ADFI), gain to feed ratio (G:F) and faecal score (FS) are presented in Table 1. There was no difference in ADG, ADFI or G:F during the 14-day experimental period. For the duration of the experiment, pigs supplemented with 250 ppm fucoidan had lower faecal scores compared with the basal group (*p* < 0.05).

### 2.2. Small Intestinal Morphology 

Villus height and crypt depth were measured in the three segments of the small intestine to evaluate the effect of fucoidan supplementation on intestinal morphology, as described in the materials and methods. Supplementation with 250 ppm fucoidan had no effect on small intestinal morphology in either the duodenum, jejunum or ileum (data presented in Table 2).

### 2.3. Large Intestinal Microbiota and Volatile Fatty Acids (VFA)

The effect of 250 ppm fucoidan supplementation on the large intestinal microbiota was determined using next-generation sequencing of the 16S rRNA gene, using the Illumina MiSeq platform as detailed in the materials and methods. Bioinformatic analysis, as described in the materials and methods, allowed for the identification of 975 OTUs. The full 16S rRNA microbial analysis data for both the caecum and colon are presented in the Supplementary Materials (Supplementary Document 1 and Supplementary Tables S1–S2). 

#### 2.3.1. Bacterial Richness and Diversity Analysis

Supplementation with 250 ppm fucoidan had no effect on the observed, Shannon or Simpson measures of alpha diversity (*p* > 0.10) (Supplementary Document 1, Figure S1). In relation to beta diversity, pigs did not cluster based on diet nor region of the large intestine (data not shown). As beta diversity is a measure of between animal variation, this suggests that large variation exists between individuals within treatments in this study.

#### 2.3.2. Differential Abundance Analysis

The differential abundances of bacterial taxa at phylum, family, genus and species level are presented as percentages in Supplementary Table S1 (caecal digesta) and Supplementary Table S2 (colonic digesta). 

The effect of 250 ppm fucoidan supplementation on the bacterial phyla is presented in Table 3. Bacteroidetes were predominant in both the caecum and colon (~50%–56%), followed by Firmicutes (~26%–30%) and Proteobacteria (~12%–19%). Dietary treatment did not influence the relative abundance of any phylum (*p* > 0.05). Genus-level analysis revealed that *Prevotella* and *Campylobacter* were the predominant genera in both the caecal (Table 4) and colonic digesta (Supplementary Table S2). There were no differences in the relative abundance of any OTU in the colon. Within the phylum Firmicutes, four differentially abundant OTUs were identified in the caecum between the basal and 250 ppm fucoidan groups. One OTU assigned to the genus *Turicibacter* (368490) (*p* < 0.01; Table 4), one OTU assigned to the family *Lachnospiraceae* (846477) and two others within the class Clostridia (358439, 555945) were reduced in fucoidan-supplemented pigs (*p* < 0.05; Table 5). Within the phylum Proteobacteria, one OTU assigned to the genus *Helicobacter* (311173) was increased in the caecal digesta of pigs supplemented with 250 ppm fucoidan (*p* < 0.01; Table 4). Within the phylum Bacteroidetes, four differentially abundant OTUs were identified in the caecum. One OTU assigned to the genus *Prevotella* (261240) was reduced, while one was assigned to the genus *Parabacteroides* (28974), one assigned to the family *RF16* (new feference OTU3588) and one which could not be assigned to any family (299713), were increased in pigs supplemented with 250 ppm fucoidan (*p* < 0.05; Table 5). 

#### 2.3.3. Selected Microbial Populations in the Caecal and Colonic Digesta

The effect of supplementation with 250 ppm fucoidan on the populations of selected microbial species in the caecum and colon were measured using QPCR; the results are presented in Table 6. Dietary supplementation with 250 ppm fucoidan had no effect on the numbers of *Bifidobacterium* spp., *Lactobacillus* spp., *Enterobacteriaceae* or total bacteria in either the caecum or colon. 

#### 2.3.4. VFA

VFA concentrations were measured in both the caecal and colonic digesta, as described in the materials and methods, to determine the effect of supplementation with 250 ppm fucoidan on microbial fermentation in the large intestine. The concentrations of the measured VFA are presented in Table 7. Fucoidan inclusion at 250 ppm had no effect on VFA in the caecum. In the colon, fucoidan supplementation increased the concentration of total VFA (*p* < 0.05), propionate (*p* < 0.01) and valerate (*p* < 0.05). Fucoidan supplementation also exhibited a tendency to increase butyrate (*p* < 0.10) concentration.

### 2.4. Gene Expression

The Nanostring nCounter was employed to examine the effect of fucoidan on the expression of genes related to intestinal health and functionality. The expression profile of 32 genes in the small intestine and 53 genes in the large intestine were measured, as described in the materials and methods. The genes that were differentially expressed are presented in Table 8, with all gene expression data presented in the Supplementary Materials (Supplementary Document 1, Tables S1–S4).

#### Digestive Enzyme and Nutrient Transporter Gene Expression

In the duodenum, fucoidan supplementation at 250 ppm upregulated sodium monocarboxylate cotransporter (*SLC5A8*; *p* < 0.05). In the jejunum, the largest fold changes (*FC*) were observed in response to fucoidan supplementation which downregulated peptide transporter 1 (*SLC15A1*; *p* = 0.05, *FC* = 1.9), sodium glucose cotransporter 1 (*SLC5A1*; *p* < 0.01, *FC* = 2.9) and sucrase-isomaltase (*SI*; *p* < 0.05, *FC* = 2.4). In the ileum, fucoidan supplementation at 250 ppm downregulated fatty acid binding protein 2 (*FABP2*; *p* < 0.05, *FC* = 1.6) and *SLC5A1* (*p* < 0.05, *FC* = 1.7).

### 2.5. Immune Marker, Tight Junctions and Transcription FactorGene Expression

In the duodenum, fucoidan supplementation at 250 ppm downregulated the tight junction gene claudin-5 (*CLDN5*; *p* < 0.05), and in the ileum, fucoidan supplementation reduced the expression of tight junction protein 1 (*TJP1*; *p* < 0.05). 

In the colon, fucoidan supplementation downregulated the expression of TNF receptor associated factor 3 (*TRAF3*; *p* < 0.05) and retinoic acid inducible gene 1 (*DDX58*; *p* < 0.05).

## 3. Discussion

In this study, we hypothesised that the optimum inclusion level of fucoidan from a 44% fucoidan extract from the species *Ascophyllum nodosum* would favourably enhance the large intestinal microbiota and reduce the incidence of post-weaning diarrhoea. For the duration of this study, faecal scores of all treatment groups remained within a healthy range, which is likely due to the good hygiene conditions and husbandry practices often observed in research facilities compared with commercial farms [22]. However, supplementation with 250 ppm fucoidan did result in a significant improvement in faecal scores. These healthier faecal scores, in association with greater concentrations of VFAs in the colon, suggest that these fucoidan-supplemented pigs had a healthier digestive tract. While it had been anticipated that the fucoidan rich extract may negatively impact performance, neither inclusion level significantly influenced growth performance; in fact, pigs supplemented with 250 ppm fucoidan had numerically higher ADG, ADFI and G:F. Despite the reduced expression of some nutrient transporters in the small intestine with the inclusion of 250 ppm fucoidan, this was not associated with any disimprovements in small intestinal morphology or growth performance. These results indicate that dietary inclusion of 250 ppm fucoidan from a 44% purified fucoidan extract from *Ascophyllum nodosum* can improve faecal consistency in pigs during the post-weaning period. Further studies to ascertain the ability of this extract to prevent post-weaning diarrhoea in pigs reared in more challenging hygiene or husbandry conditions are warranted. 

The main hypothesis of this study is that fucoidan supplementation would enhance the large intestinal microbiota. Dietary supplementation with fucoidan previously increased lactobacilli numbers in the faeces [14,15] and also in the proximal and distal colon [13] of pigs. Surprisingly, while faecal consistency was improved and colonic VFA concentrations were increased, fucoidan supplementation had no effect on the colonic microbiota in this study. These contrasting responses to fucoidan supplementation may be related to the species of seaweed from which the fucoidan was derived. *Ascophyllum nodosum* was used in this study while the aforementioned studies in which increases in lactobacilli were observed used fucoidan derived from *Laminaria* spp. Fucoidans can be classified into two groups, those with long chains of (1→3)-linked α-l-fucopyranosyl as found in *Laminaria* spp., and those with alternating (1→3)- and (1→4)-linked α-l-fucopyranosyl residues [8] found in *Ascophyllum nodosum* and *Fucus* spp. Thus, these differing structures may explain the varying responses to fucoidan supplementation among studies. However, beyond species differences, the biological activities of the extract can also differ depending on the season of harvest and also due to the extraction methodology and conditions employed such as solvent, pH, time and pressure [18]. Previously, the methods used for measuring the bacterial populations varied from traditional culture methods to QPCR compared with 16S rRNA sequencing used in this study; this difference may also have contributed to the differing outcomes. 

Both QPCR and 16S rRNA sequencing were utilized to analyse the effects of fucoidan on the large intestinal bacterial community. In the caecum, fucoidan had no effect on the relative bacterial abundance at phylum, class, family or species level. Bacteroidetes, Firmicutes and Proteobacteria were the predominant phyla observed in both groups of pigs, and these have previously been identified as the predominant phyla in both suckling and weaned pigs [23,24]. Consistent with previous reports, *Prevotella* was identified as the predominant genus in both groups; this genus is associated with the introduction of a plant-based diet due to their ability to degrade hemicelluloses such as xylans present in plants [23,24]. One OTU assigned to the genus *Prevotella* was reduced in fucoidan-supplemented pigs; however, other OTUs assigned to this genus were not affected. Fucoidan also reduced the relative abundance of OTUs within the phylum Firmicutes, two assigned to the class clostridia and one assigned to the family *Lachnospiraceae*. This family is associated with the production of butyrate, in particular, *Roseburia* spp. Species belonging to *Lachnospiraceae* can convert lactate into butyrate [25]. While there were no changes in the relative abundance of bacterial communities within the colon, the concentration of propionate, valerate, butyrate and total VFA were higher in pigs supplemented with fucoidan. Propionate is produced from highly fermentable carbohydrates and sugars, acetate and butyrate are associated with the fermentation of fibre [26], and valerate is formed through the fermentation of undigested/unabsorbed protein or endogenous protein [27]. VFA are involved in the maintenance of colonic homeostasis; in particular, butyrate is the preferential energy source of the colonocytes accounting for about 70% of total energy consumption [27]. VFA also have antidiarrheal effects as they promote the absorption of sodium and water [27,28]; thus, the increased VFA concentrations may be related to the improved faecal scores in the pigs supplemented with fucoidan. 

Supplementation with 250 ppm fucoidan downregulated the gene expression of some digestive enzymes and nutrient transporters in the small intestine. This group had a 2.4-fold reduction in *SI*, an enzyme complex involved in the final digestion of disaccharides and oligosaccharides to absorbable monosaccharides. *SI* expression was previously shown to be upregulated in the rat jejunum following a sucrose diet suggesting its expression is regulated by dietary carbohydrates [29]. Similar to this reduction in *SI*, in vitro fucoidans from *Ascophyllum nodosum* were shown to suppress α-amylase (salivary) and α-glucosidase [30]. The ability of fucoidan to reduce α-amylase was shown to be dependent on its molecular weight and degree of sulphation [31]. Following digestion, nutrient transporters enable the transfer of digestion products (monosaccharides, peptides, amino acids and fatty acids) from the lumen into the enterocytes [32]. In this study the expression of *SLC5A1*/*SGLT1* (jejunum and ileum) which transports glucose [33], *SLC15A1*/*PEPT1* (jejunum) which transports di- and tri-peptides [34] and *FABP2* (ileum) which transports long chain fatty acids [35] were all downregulated (2.9-,1.7-, 1.9-, 1.6- fold, respectively) following fucoidan supplementation. The reduction in the gene expression of digestive enzymes and nutrient transporters may be due to the presence of both fucoidan and alginate in the extract. Fucoidan is a non-digestible polysaccharide in the upper gastrointestinal tract [36], and by increasing digesta viscosity, it may disrupt the flow of digesta, reducing the mixing of digesta with digestive fluids. Similarly, alginate is a viscous soluble fibre which can delay gastric emptying through the formation of gels within the stomach and affect the rheological properties of the digestive contents [37]. As the gene expression of nutrient transporters can be modified by fluctuations in available nutrients [38], it is possible the presence of fucoidan and/or alginate within the intestine may have led to the downregulation of nutrient transporters in the supplemented group. Unfortunately, ileal digestibility’s could not be measured in this study due to a lack of digesta in the ileum at the time of sampling. This may have enhanced our understanding of the effects of the various components of the fucoidan-rich extract on nutrient digestion and absorption as the changes in gene expression were not coupled with changes in villus architecture or growth performance. Perhaps there are other mechanisms at play which have offset the effects of the downregulated nutrient transporter genes in terms of overall growth performance. 

The pig is a commonly used model for studying the effects of dietary supplements within the gastrointestinal tract due to its anatomical, physiological and functional similarities with humans [39]. As fucoidan is widely investigated for use in the prevention/treatment of metabolic syndromes, including obesity and diabetes (Wang, et al. [40]), the results from this study may provide relevant information for future studies. In this study, the fucoidan rich extract reduced the ileal expression of *FABP2*. Similarly, fucoidan reduced the expression of fatty acid binding protein 4 (FABP4) in vitro in 3T2-L1 adipocytes [41], suggesting that fucoidan has the potential to reduce fatty acid absorption in different cell types. Fucoidan-supplemented pigs also had increased colonic propionate. Previously, propionate increased the secretion of the appetite-regulating hormones PYY and GLP-1 [42] in-vitro in cultured colonic cells [42]. Furthermore, short term dietary supplementation with an inulin propionate ester increased PYY and GLP-1 secretion in the colon and reduced feed intake, while long term supplementation reduced weight gain and intra-abdominal fat deposition in overweight adults [42]. It has also been suggested that fucoidan can influence glucose metabolism [40]. As mentioned above, the gene expression of *SI* and *SGLT1* were downregulated in pigs supplemented with a fucoidan-rich extract, indicating fucoidan may have the potential to reduce the accessibility of dietary carbohydrates. This aligns with the aforementioned in-vitro reduction of α-amylase and α-glucosidase with fucoidan derived from *Ascophyllum nodosum*, while fucoidan derived from *Fucus vesiculosus* only inhibited α-glucosidase [30], suggesting *Ascophyllum nodosum* is a better source of fucoidan for the prevention of Type 2 diabetes. Ganesan et al. [43] suggested the inhibitory activities of fucoidan on glucose metabolism may be related to the interaction between the negatively charged sulphate groups of fucoidan and digestive enzymes or may be related to the high viscosity of fucoidan influencing the accessibility of nutrients to digestive enzymes. Thus, the reduced gene expression of fatty acid and glucose transporters, digestive enzymes and increased colonic propionate suggest fucoidan warrants further study as a dietary supplement for the prevention or treatment of metabolic diseases such as obesity and diabetes. 

## 4. Materials and Methods 

All experimental procedures described in this work were approved under the University College Dublin Animal Research Ethics Committee (AREC-17-19-O’Doherty) and were conducted in accordance with Irish legislation (*SI* no. 543/2012) and the EU directive 2010/63/EU for animal experimentation. 

### 4.1. Experimental Design and Diets 

This experiment comprised 3 dietary treatments: (T1) basal diet; (T2) basal diet + 125 ppm fucoidan; (T3) basal diet + 250 ppm fucoidan. Previously, fucoidan demonstrated beneficial effects at an inclusion level of 240 ppm [9,13,14]; however, its effects at lower inclusion levels were unknown. Thus, the fucoidan dietary treatments were formulated to contain either 125 or 250 ppm fucoidan. Seventy-two healthy piglets (progeny of meatline boars × (large white × landrace sows)) with an average weaning weight of 8.4 kg (SD 1.06) were sourced from a commercial farm at weaning (28 days of age) and housed in pens of three. The pigs were blocked based on weaning weight, the litter of origin and sex and, within each block, assigned to one of the three dietary treatments (eight replicates/treatment). The basal diet contained 14.95 MJ/kg digestible energy, 190 g/kg crude protein (CP) and 13.5 g/kg total lysine. All amino acid requirements were met relative to lysine [44]. The ingredient and chemical analysis of the dietary treatments is presented in Table 9. The fucoidan rich extract was a commercial product sourced from BioAtlantis Ltd (Clash Industrial Estate, Tralee, Co. Kerry, Ireland). A single extraction was performed from *Ascophyllum nodosum* to produce the commercial product which contained 441 g of fucoidan per kg DM, 25.9 g laminarin/kg DM, 135 g alginates/kg DM, 43.8 g mannitol/kg DM, 34.8 g phlorotannins/kg DM and 319.5 g ash/kg DM. The appropriate quantity of the fucoidan rich extract was added to the basal diet to achieve 125 or 250 ppm fucoidan inclusion levels. 

### 4.2. Housing and Animal Management 

The pigs were housed in fully slatted pens (1.7 × 1.2 m). Pigs were weighed at the beginning of the experiment (d0; day of weaning) and on days 7 and 14. The ambient environmental temperature within the house was thermostatically controlled at 30 °C for the first 7 days and then reduced by 2 °C for the remainder of the second week, and the humidity was maintained at 65%. Feed in meal form and water were available ad libitum from four-space feeders and nipple drinkers; precaution was taken to avoid wastage of feed. Everyday throughout the experiment, faecal scores were recorded in the individual pens by the same operator on a scale ranging from 1 to 5 as follows: 1 = hard, firm faeces; 2 = slightly soft faeces; 3 = soft, partially formed faeces; 4 = loose, semi-liquid faeces; and 5 = watery, mucous-like faeces [14]. 

### 4.3. Sample Collection

On day 15, eight pigs (one pig/pen) from the basal group and best performing fucoidan treatment (250 ppm) group (based on FS) received a lethal injection with pentobarbitone sodium (euthatal solution, 200 mg/mL; Merial Animal Health, Essex, UK) at a rate of 0.71 mL/kg BW to the cranial vena cava to humanely sacrifice the animals. Euthanasia was completed by a trained individual in a separate room from the other pigs. The entire intestinal tract was removed immediately. Sections from the duodenum (10 cm from the stomach), the jejunum (60 cm from the stomach) and the ileum (15 cm from the caecum) were excised and fixed in 10% phosphate-buffered formalin. Digesta from the caecum and colon was collected in sterile containers (Sarstedt, Wexford, Ireland) and frozen immediately for further analysis. In addition, tissue samples were taken from the duodenum, jejunum, ileum and colon to establish relative gene expression of a range of functional categories, including cytokines, digestive enzymes, nutrient transporters, mucins, tight junction components, pathogen recognition receptors, transcription regulators, appetite regulators, growth factors, kinases, ligand-dependent nuclear receptors, suppressors of cytokine signalling, peptidases, transmembrane receptors and viral defence genes. Relative gene expression was measured using the Nanostring nCounter. Tissue sections of 1 cm^2^ from the duodenum, jejunum, ileum, and colon were excised, emptied by dissecting them along the mesentery and rinsed using sterile PBS (Oxoid, Hampshire, UK). The tissue sections were stripped of overlying smooth muscle and stored in 5 mL RNAlater^®^ solution (Applied Biosystems, Foster City, CA, USA) overnight at 4 °C. The RNAlater^®^ was then removed before storing the samples at −80 °C. 

### 4.4. Feed Analysis

The feed samples were milled through a 1 mm screen (Christy and Norris hammer mill, Ipswich, UK). The dry matter (DM) of the feed was determined after drying overnight at 104 °C. Crude ash content was determined after the ignition of a known weight of concentrate in a muffle furnace (Nabertherm, Bremen, Germany) at 550 °C for 6 h. The crude protein (CP) content was determined as Kjeldahl N × 6.25 using the LECO FP 528 instrument. The neutral detergent fibre (NDF) content was determined according to Van Soest et al. [46]. 

### 4.5. Gut Morphological Analysis

Preserved duodenal, jejunal and ileal tissue samples were prepared using standard paraffin-embedding techniques. The samples were sectioned at a thickness of 5 μm and stained with haematoxylin and eosin. Villus height (VH) and crypt depth (CD) were measured in the stained sections (4 × objective) using a light microscope fitted with an image analyser (Image-Pro Plus; Media Cybernetics, Oxon, UK. Measurements of 15 correctly orientated and intact villi and crypts were taken for each segment. The VH was measured from the crypt-villus junction to the tip of the villus, and CD was measured from the crypt-villus junction to the base. Results are expressed as mean VH or CD in μm. 

### 4.6. Gene Expression

#### 4.6.1. RNA Extraction

Total RNA was extracted from duodenal, jejunal, ileal and colonic tissue using TRIreagent (Sigma-Aldrich, St. Louis, MS, USA) according to the manufacturer’s instructions. The crude RNA extract was further purified using the GenElute Mammalian Total RNA miniprep kit (Sigma-Aldrich) according to the manufacturer’s instructions. A DNase step was included using an on-Column Dnase 1 digestion set (Sigma-Aldrich, St. Louise, MS, USA). The total RNA was quantified using the Nanodrop-ND1000 spectrophotometer (Thermo Fisher Scientific, Wilmington, USA) and purity was assessed by determining the ratio of the absorbance at 260 and 280 nm. All total RNA samples had 260:280 nm ratios above 2.0.

#### 4.6.2. Nanostring nCounter Analysis

The small intestinal (duodenal, jejunal and ileal) tissues and colonic tissue were analysed using the Nanostring nCounter analysis system (Nanostring Technologies, Seattle, WA, USA). Two custom nCounter panels, one for the small intestine and one for the colon were designed by our group and manufactured by Nanostring (Nanostring Technologies, Seattle, USA). The panel for the small intestine is presented in Table 10 and containes 32 target genes and 5 reference genes. The genes measured in the colon are presented in Table 11; this codeset contained 53 target genes and 8 reference genes. Both panels contained 6 positive and 8 negative controls. 

The expression of all target genes was determined for each sample in a single multiplexed hybridisation reaction, as originally described by Geiss et al. [47]. Briefly, prior to analysis, all samples were measured using the Qubit fluorometer (Thermo Fisher Scientific, Wilmington, USA) and calibrated to 20 ng/μL. For the hybridisation reaction, a master mix (MM) was created by adding 70 μL of hybridisation buffer to the reporter codeset, as per manufacturer instructions. To each reaction tube, 8 μL of MM, 5 μL of sample (total RNA concentration 100 ng) and 2 μL capture probeset were added and inverted to mix, then centrifuged briefly before incubation at 65 °C for 20 h in a Bio-rad thermocycler (Bio-rad Laboratories Ltd., Watford, Hertfordshire, UK). Post-hybridisation processing was performed within the Nanostring nCounter prep station (Nanostring Technologies, Seattle, USA); this liquid handling system removes excess unbound probes and immobilises samples onto the internal surface of the sample cartridge. Following this, the cartridge is sealed and scanned in the digital analyser (Nanostring Technologies, Seattle, USA), which collects data from the immobilized fluorescent reporters in the sample cartridge. The analysis and normalisation of the raw Nanostring data was performed using nSolver analysis software v4.0 (Nanostring Technologies). Background was corrected for using background thresholding, and a background count level was estimated using the average count of the negative control probes in every reaction plus two standard deviations [48]. Target genes with raw counts below the threshold in more than two-thirds of samples were excluded from the analysis. Raw counts were normalised using a combination of positive control normalisation and CodeSet content normalisation. The former accounts for errors such as pipetting errors, lot-to-lot variation in nCounter preparation plates and nCounter cartridges, while the latter uses housekeeping genes to account for variability in the quantity and quality of sample RNA. Nanostring results (raw and normalised counts) were produced from RCC files using nSolver software v 4.0.

### 4.7. Microbiological Analyses

#### 4.7.1. Microbial DNA Extraction 

Microbial genomic DNA was extracted from the caecal and colonic digesta samples using a QIAamp DNA stool kit (Qiagen, West Sussex, UK) in accordance with the manufacturer’s instructions. The quantity and quality of DNA were assessed using a Nanodrop spectrophotometer (Thermo Fisher Scientific, Wilmington, DE, USA).

#### 4.7.2. Illumina Sequencing

High-throughput sequencing of the V3-V4 hypervariable region of the bacterial 16S rRNA gene was performed on an Illumina MiSeq platform according to their standard protocols (Eurofins, Wolverhampton, UK). Briefly, the V3-V4 region was PCR-amplified using universal primers containing adapter overhang nucleotide sequences for forward and reverse index primers. Amplicons were purified using AMPure XP beads (Beckman Coulter, Indianapolis, IN, USA) and set up for the index PCR with Nextera XT index primers (Illumina, San Diego, CA, United States). The indexed samples were purified using AMPure XP beads, quantified using a fragment analyzer (Agilent, Santa Clara, CA, USA), and equal quantities from each sample were pooled. The resulting pooled library was quantified using the Bioanalyzer 7500 DNA kit (Agilent) and sequenced using the v3-v4 chemistry (2 × 300 bp paired-end reads). 

#### 4.7.3. QPCR

Quantitative PCR (QPCR) was used to validate the sequencing results for the following bacterial groups: *Bifidobacterium* spp., *Lactobacillus* spp., *Enterobacteriaceae* and total bacteria. For the QPCR, standard curves were prepared with pooled aliquots of caecal and colonic digesta DNA, as described previously [49]. Domain, genus and family specific primers are presented in Table 12. The selected bacterial groups were estimated based on gene copy number (GCN) in the digesta using QPCR on the 7500 Fast Real-Time PCR system (Applied Biosystems). QPCR was carried out in a final reaction volume of 20 μL containing 3 μL template DNA, 1 μL of forward and reverse primers (100 pM), 10 μL SYBR Green PCR master mix (Applied Biosystems) and 5 μL nuclease-free water. The thermal cycling conditions involved an initial denaturation step at 95 °C for 10 min, followed by 40 cycles of 95 °C for 15 s and 65 °C for 1 min. Dissociation curves confirmed the specificity of the final PCR products. All samples were prepared in duplicate, and the mean threshold cycle (Ct) value was used for calculations. The estimates of GCN for selected bacteria were log-transformed and are presented as GCN per gram of digesta.

### 4.8. VFA 

Digesta from the caecum and colon was collected to determine VFA concentrations using gas–liquid chromatography according to the method described by Pierce et al. [50]. A 1 g sample was diluted with distilled water (2.5 × weight of sample) and centrifuged at 1400× *g* for 10 min (Sorvall GLC–2 B laboratory centrifuge, DuPont, Wilmington, DE, USA). One mL of the subsequent supernatant and 1 mL of internal standard (0.05% 3-methyl-*n*-valeric acid in 0.15 M oxalic acid dihydrate) were mixed with 3 mL of distilled water. The reaction mixture was centrifuged at 500× *g* for 10 min, and the supernatant was filtered through 0.45 PTFE (polytetrafluoroethylene) syringe filter into a chromatographic sample vial. An injection volume of 1 μL was injected into a Varian 3800 GC equipped with an EC™ 1000 Grace column (15 m × 0.53 mm I.D) with 1.20 μm film thickness. The temperature programme set was 75–95 °C increasing by 3 °C/minute, 95–200 °C increasing by 20 °C/minute, which was held for 0.50 min. The detector and injector temperature were 280 and 240 °C, respectively, while the total analysis time was 12.42 min.

### 4.9. Bioinformatic and Statistical Analyses

The resulting sequences were analysed using the open source software package Quantitative Insights into Microbial Ecology (Qiime) [51]. Initially, sequencing primers were removed using the cutadapt function of Qiime. Paired-end reads were then joined with the multiple join paired-end reads function within Qiime using the default parameters. Using the split libraries function, the raw reads were initially demultiplexed, and reads were quality filtered using default QIIME parameters and sequences that contained ambiguous characters, non-exact barcode matches, sequence length <225 nucleotides and having a read-quality score of <27 were removed. OTUs were picked at 97% sequence similarity using the uclust function within Qiime [51,52]. Singletons were removed, as only OTUs that were present at the level of at least two reads in more than one sample were retained. The resulting OTU representative sequences were assigned to different taxonomic levels (from phylum to species) using the GreenGenes database. Chimeras were identified and removed with the use of ChimeraSlayer [53,54]. The normalized OTU table combined with the phenotype metadata and phylogenetic tree comprised the data matrix. This matrix was then input into the phyloseq package within the R (http://www.r-project.org; version 3.5.0). The dynamics of richness and diversity in the piglet’s microbiota were computed with the observed, the Simpson and the Shannon indices. The Simpson and Shannon indices of diversity account for both richness and evenness parameters. To estimate beta diversity measurements, which are a measure of separation of the phylogenetic structure of the OTU in one sample compared with all other samples, the data was normalised to make taxonomic feature counts comparable across samples. Several distance metrics were considered, in order to calculate the distance matrix of the different multidimensional reduction methods. These included weighted/unweighted UniFrac distance and non-phylogenetic distance metrics (i.e., Bray–Curtis, Jensen–Shannon divergence and Euclidian) using phyloseq in R [55,56]. Taxonomy and diversity plots were produced using graphics tailored for phylogenetic analysis using the R package ggplot2 [57]. Differential abundance testing was performed using the phyloseq to deseq2 function within R [56,58]. Results are presented using Benjamini–Hochberg (BH) adjusted *p*-values. 

All other data were initially checked for normality using the univariate procedure of Statistical Analysis Software (SAS) 9.4 (SAS Institute, Cary, NC, USA). The performance data and FS data were analysed using repeated measures within the mixed procedure of SAS, and the model included fixed effects of treatment, time and their associated interactions. The initial weight was used as a covariate for the performance data. The data on intestinal morphology, microbial populations, gene expression and volatile fatty acids were analysed using the GLM procedure of SAS. The model assessed the effect of treatment, with the pig being the experimental unit. The probability level that denoted significance was *p* < 0.05, while *p*-values between 0.05 and 0.1 are considered numerical tendencies. Data are presented as least-square means with their standard errors of the mean.

## 5. Conclusions

The improved faecal consistency observed in fucoidan-supplemented pigs was likely related to increased water absorption associated with the increased VFAs in the colon. Despite the reduced expression of genes involved in nutrient digestion and transport, this extract did not negatively impact growth performance or small intestinal morphology. Thus, fucoidan derived from *A. nodosum* at 250 ppm warrants further study for use as a dietary supplement to prevent post-weaning diarrhoea in more challenging conditions such as those observed on commercial farms. This fucoidan-rich extract also demonstrated effects on the gene expression of digestive enzymes, nutrient transporters and an increase in colonic propionate, effects which suggest it merits further investigation as a dietary supplement for the prevention or treatment of metabolic diseases. 

## Figures and Tables

**Table 1:** Effect of increasing fucoidan inclusion level on pig growth performance and faecal consistency (least square means with their standard errors).

	Fucoidan Inclusion Level (PPM) *			SEM	Time (day)		SEM	*p*-Values		
	0	125	250		7	14		Treatment	Time	Treatment × Time
ADG (kg)	0.160	0.130	0.178	0.020	0.080	0.228	0.016	0.259	<0.001	0.863
ADFI (kg)	0.319	0.308	0.340	0.011	0.209	0.435	0.009	0.104	<0.001	0.378
G:F	0.467	0.403	0.517	0.060	0.391	0.533	0.049	0.430	0.042	0.846
FS	2.97 ^a^	2.94 ^a,b^	2.72 ^b^	0.080	2.801	2.953	0.065	0.038	0.108	0.765

ADG, average daily gain; ADFI, average daily feed intake; G:F, gain to feed ratio; FS, faecal score; d, days; ^a,b^ Mean values within a row with unlike superscript letters were significantly different (*p* < 0.05). * A total of eight replicates were used per treatment group (replicate = pen, 3 pigs/pen).

**Table 2:** Effect of 250 ppm fucoidan on villus height and crypt depth in the small intestine (least square means with their standard errors).

	Basal *	Fucoidan 250 ppm *	SEM	*p*-Value
**Duodenum**				
VH μm	219.32	228.61	49.58	0.696
CD μm	112.88	125.20	21.14	0.234
VH:CD	1.95	1.86	0.43	0.660
**Jejunum**				
VH μm	212.42	231.87	48.15	0.404
CD μm	135.27	149.62	32.71	0.366
VH:CD	1.59	1.60	0.36	0.928
**Ileum**				
VH μm	242.43	249.74	49.90	0.760
CD μm	125.68	121.59	26.77	0.750
VH:CD	1.98	2.11	0.49	0.602

VH, villus height; CD, crypt depth; VH:CD, villus height to crypt depth ratio; * a total of 8 replicates were used per treatment group.

**Table 3:** Effect of 250 ppm fucoidan on the relative abundance of bacterial phyla in the caecal and colonic digesta (mean % relative abundance with their standard errors).

	Caecum				Colon			
Phylum	Basal *	Fucoidan 250 ppm *	SEM	Adjusted *p*-Value	Basal *	Fucoidan 250 ppm *	SEM	Adjusted *p*-Value
Bacteroidetes	50.82	54.23	2.28	0.277	54.58	56.08	2.47	0.996
Firmicutes	27.58	26.87	2.03	0.246	29.57	28.76	2.82	0.996
Proteobacteria	19.44	16.71	2.55	0.246	12.08	12.43	2.30	0.996
Spirochaetes	1.53	1.43	0.49	0.859	2.49	1.83	0.57	0.670
Deferribacter	0.21	0.18	0.06	0.859	0.61	0.42	0.21	0.996
Fusobacteria	0.20	0.12	0.11	0.859	0.31	0.00	0.15	0.670
Tenericutes	0.10	0.34	0.07	0.358	0.18	0.29	0.06	0.996
Actinobacteria	0.07	0.08	0.01	0.859	0.09	0.09	0.02	0.996
Fibrobacteres	0.04	0.04	0.01	0.859	0.09	0.10	0.04	0.996

* A total of 8 replicates were used per treatment group.

**Table 4:** Effect of 250 ppm fucoidan on the relative abundance of bacterial genera in the caecal digesta (mean % relative abundance with their standard errors).

Genus	OTU	Basal *	Fucoidan 250 ppm *	SEM	Adjusted *p*-Value
*Prevotella*	568118	42.73	43.91	3.49	0.911
*Campylobacter*	113756	14.02	13.94	2.20	0.888
[*Prevotella*]	20534	11.98	11.92	1.56	0.797
*Roseburia*	New.CleanUp.ReferenceOTU122441	5.23	5.26	1.38	0.985
*Lactobacillus*	302975	3.71	2.57	1.07	0.797
*Faecalibacterium*	851865	3.30	2.41	0.79	0.911
*Anaerovibrio*	New.ReferenceOTU1058	2.44	2.44	0.69	0.888
*Treponema*	68837	1.98	1.93	0.63	0.901
*Bacteroides*	New.ReferenceOTU2302	1.95	1.34	0.96	0.614
*CF231*	300853	1.89	3.00	0.41	0.911
*Oscillospira*	310886	1.87	2.29	0.21	0.955
*Succinivibrio*	163857	1.80	0.89	0.76	0.183
*Actinobacillus*	359779	1.50	0.73	0.57	0.614
*Lachnospira*	843553	0.94	0.59	0.20	0.614
*Parabacteroides*	28974	0.79	1.58	0.37	0.911
*Coprococcus*	1107057	0.69	0.58	0.13	0.708
*Clostridium*	215963	0.42	0.32	0.10	0.880
*YRC22*	4435235	0.37	0.55	0.14	0.968
*Ruminococcus*	148925	0.35	0.35	0.06	0.888
*Fusobacterium*	1654477	0.31	0.23	0.18	0.797
*Mucispirillum*	4374042	0.27	0.23	0.07	0.911
*Turicibacter*	368490	0.21	0.00	0.08	0.002
*Sutterella*	333380	0.18	0.29	0.04	0.911
*Blautia*	696563	0.12	0.11	0.02	0.911
*Dorea*	1076587	0.09	0.13	0.02	0.797
*Mitsuokella*	149335	0.08	0.06	0.03	0.911
*Desulfovibrio*	30569	0.08	0.48	0.11	0.183
*Butyrivibrio*	4364564	0.08	0.03	0.03	0.797
*Streptococcus*	349024	0.07	0.02	0.02	0.507
*Helicobacter*	311173	0.07	0.80	0.18	0.002
*Aggregatibacter*	9498	0.06	0.00	0.03	0.797
*Anaerovorax*	1112364	0.05	0.01	0.02	0.888
*Megasphaera*	266210	0.05	0.06	0.02	0.985
*Fibrobacter*	New.ReferenceOTU3654	0.05	0.05	0.02	0.911
*Phascolarctobacterium*	916143	0.05	0.05	0.01	0.955
[*Ruminococcus*]	1111191	0.04	0.00	0.02	0.614
*Anaeroplasma*	New.ReferenceOTU3606	0.04	0.22	0.07	0.911
*Epulopiscium*	New.ReferenceOTU2736	0.03	0.01	0.01	0.911
*Collinsella*	363794	0.03	0.06	0.01	0.614
*Anaerobiospirillum*	587570	0.03	0.40	0.10	0.183
*rc4-4*	New.ReferenceOTU2707	0.02	0.03	0.01	0.911
*Anaerostipes*	New.ReferenceOTU1761	0.01	0.01	0.00	0.708
*Slackia*	367139	0.00	0.02	0.01	0.614
*Acidaminococcus*	25947	0.00	0.01	0.00	0.593
*Oxalobacter*	360508	0.00	0.01	0.00	0.797
*Bilophila*	New.ReferenceOTU2103	0.00	0.01	0.00	0.481
*Dialister*	264552	0.00	0.04	0.01	0.221
*Mycoplasma*	1143674	0.00	0.07	0.03	

OTU, operational taxonomic unit; * A total of 8 replicates were used per treatment group.

**Table 5:** Differentially abundant OTUs in the caecum of pigs fed a basal diet supplemented with 250 ppm fucoidan. A negative log2FoldChange indicates a reduction, while a positive log2FoldChange indicates an increase in abundance in the 250 ppm fucoidan group compared to the basal group (n = 8/treatment).

	OTU	BaseMean	Log2FoldChange	lfcSE	Stat	Adjusted *p*-Value
**Class**						
Clostridia	358439	88.53	−3.432	1.040	−3.299	0.039
Bacteroidia	299713	267.65	7.458	2.119	3.520	0.039
Clostridia	555945	33.80	−4.596	1.327	−3.464	0.039
**Family**						
Lachnospiraceae	846477	50.79	−3.815	1.182	−3.227	0.039
RF16	New.ReferenceOTU3588	309.62	3.792	1.128	3.362	0.039
**Genus**						
Prevotella	261240	246.06	−4.086	1.255	−3.256	0.039
Parabacteroides	28974	311.19	3.027	0.942	3.215	0.039
Turicibacter	368490	16.56	−4.215	1.171	−3.599	0.039

OTU, operational taxonomic unit; lfsce, logfoldchange standard error; stat, wald statistic.

**Table 6:** Effect of 250 ppm fucoidan on selected microbial populations in the caecum and colon (least square means with their standard errors).

	Basal *	Fucoidan 250 ppm *	SEM	*p*-Value
**Caecal bacterial numbers Log GCN/g digesta**				
*Bifidobacterium* spp.	6.53	6.48	0.078	0.676
*Lactobacillus* spp.	8.34	8.31	0.219	0.938
*Enterobacteriaceae*	8.30	8.08	0.277	0.613
Total bacteria	8.90	8.77	0.133	0.456
**Colonic bacterial numbers Log GCN/g digesta**				
*Bifidobacterium* spp.	5.57	5.50	0.363	0.389
*Lactobacillus* spp.	8.19	8.14	0.135	0.847
*Enterobacteriaceae*	8.32	8.14	0.295	0.684
Total bacteria	9.25	9.07	0.105	0.219

GCN, gene copy numbers. * A total of 8 replicates were used per treatment group.

**Table 7:** Effect of fucoidan inclusion of VFA in mmol/g digesta in the caecum and colon (least square means with their standard errors).

	Basal *	Fucoidan 250 ppm *	SEM	*p*-Value
**Caecal mmol/g digesta**				
Acetate	92.58	92.05	3.55	0.918
Propionate	19.85	21.71	1.28	0.322
Butyrate	12.76	12.23	1.44	0.798
Isobutyrate	0.63	0.36	0.11	0.093
Valerate	1.27	1.36	0.15	0.695
Isovalerate	0.45	0.40	0.05	0.470
Total VFA	127.55	128.11	4.73	0.935
Branched chain VFA’s	2.36	2.12	0.19	0.380
**Colonic mmol/g digesta**				
Acetate	95.36	106.06	4.85	0.146
Propionate	19.05	24.86	1.33	0.009
Butyrate	12.64	19.72	2.58	0.077
Isobutyrate	1.04	0.95	0.23	0.804
Valerate	1.72	3.25	0.37	0.012
Isovalerate	0.90	1.02	0.15	0.571
Total VFA	130.71	155.86	7.81	0.042
Branched chain VFA’s	3.66	5.23	0.62	0.097

VFA, volatile fatty acids. * A total of eight replicates were used per treatment group.

**Table 8:** Effect of supplementation with 250 ppm fucoidan on the expression of genes involved in nutrient digestion and transport in the small intestine and the expression of genes involved in immune responses and intestinal integrity in the small intestine and colon (least square means with their standard errors).

Region	Gene	Basal *	Fucoidan 250 ppm *	SEM	*p*-Value
**Nutrient transporters and digestive enzymes**					
Duodenum	*SLC5A8*	3214.00	4059.56	199.78	0.010
Jejunum	*SLC15A1*	1218.79	648.81	190.18	0.054
	*SLC5A1*	7466.46	2564.99	937.75	0.003
	*SI*	20997.41	8785.56	3261.91	0.020
Ileum	*FABP2*	13068.53	8027.51	1440.41	0.025
	*SLC5A1*	14956.02	9008.17	1924.65	0.044
**Markers of immune response and intestinal integrity**					
Duodenum	*CLDN5*	65.53	55.71	3.18	0.047
Ileum	*TJP1*	1159.5	987.10	55.73	0.044
Colon	*DDX58*	2789.66	1829.72	232.24	0.011
	*TRAF3*	152.82	130.90	7.12	0.047

*SLC5A8*, sodium monocarboxylate cotransporter 8; *SLC15A1*, peptide transporter 1; *SLC5A1*, sodium glucose cotransporter 1; *SI*, sucrase isomaltase; *FABP2*, fatty acid binding protein 2; *CLDN5*, claudin 5; *TJP1*, tight junction protein 1; *DDX58*, retinoic acid inducible gene 1; *TRAF3*, TNF receptor associated factor 3. * A total of 8 replicates were used per treatment group.

**Table 9:** Ingredient and chemical composition of basal diet *.

Ingredient (g/kg)	
Wheat	340.0
Full fat soya	170.0
Flaked wheat	130.0
Soya bean meal	105.0
Flaked maize	70.0
Whey powder	50.0
Soya oil	65.0
Vitamins and minerals ^a^	2.5
Sodium bicarbonate	2.0
Mono calcium phosphate	4.0
Calcium carbonate (Limestone)	6.0
Salt	2.0
Lysine HCL	4.0
DL-methionine	1.5
L-threonine	1.5
**Chemical analysis**	
DM	866.1
Crude protein (N × 6.25)	190
Digestible energy (MJ/kg) ^†^	14.95
Ash	48.4
Neutral detergent fibre	114.00
Lysine ^†^	13.5
Methionine and cysteine ^†^	7.4
Threonine ^†^	7.9
Tryptophan ^†^	2.6
Calcium ^†^	7.2
Phosphorous ^†^	6.0

* Treatments: (1) basal diet; (2) basal diet + 125 parts per million (ppm) fucoidan; (3) basal diet + 250 ppm fucoidan. ^†^ Calculated for tabulated nutritional composition [45]. ^a^ Provided (mg/kg complete diet): Cu, 100; Fe, 140; Mn, 47; Zn, 120; I, 0.6; Se, 0.3; retinol, 1.8; cholecalciferol, 0.025; α-tocopherol, 67; phytylmenaquinone, 4; cyanocobalamin, 0.01; riboflavin, 2; nicotinic acid, 12; pantothenic acid, 10; choline chloride, 250; thiamine, 2; pyridoxine, 0.015. Celite included at 300 mg/kg complete diet.

**Table 10:** Panel of genes analysed in the small intestine.

Group	Gene	Accession
Nutrient transporters	*SLC15A1*	NM_214347.1
	*SLC5A1*	NM_001164021.1
	*SLC2A1*	XM_003482115.1
	*SLC2A2*	NM_001097417.1
	*SLC2A5*	XM_021095282.1
	*SLC2A7*	XM_003127552.3
	*SLC2A8*	XM_003480608.1
	*FABP2*	NM_001031780.1
	*SLC16A10*	XM_021091212.1
	*SLC6A19*	XM_003359855
	*SLC7A1*	NM_001012613.1
	*SLC5A8*	NM_001291414
	*SLC16A1*	NM_001128445.1
Appetite regulators	*CCK*	NM_214237.2
	*GLP2R*	NM_001246266.1
	*GCG*	NM_214324
Digestive enzymes	*SI*	XM_021069748
	*CNDP1*	NM_001290324.1
Inflammatory markers	*NFKB1*	NM_001048232.1
	*CXCL8*	NM_213867.1
	*TGFB1*	NM_214015.2
	*IFNG*	NM_213948.1
	*IL1A*	NM_214029.1
Tight junctions	*TJP1*	XM_005659811.1
	*OCLN*	NM_001163647.2
	*CLDN3*	NM_001160075.1
	*CLDN5*	NM_001161636.1
Toll-like receptors	*TLR2*	NM_213761.1
	*TLR4*	NM_001113039.2
	*TLR5*	NM_001348771.1
Mucins	*MUC1*	XM_013997019
	*MUC2*	XM_013989745
Reference	*ACTB*	XM_003124280.4
	*B2M*	NM_213978.1
	*GAPDH*	NM_001206359.1
	*PPIA*	NM_214353.1
	*HPRT*	NM_001032376.2

*SLC15A1*, peptide transporter 1; *SLC5A1*, sodium glucose cotransporter; *SLC2A1*, glucose transporter 1; *SLC2A2*, glucose transporter 2; *SLC2A5*, glucose transporter 5; *SLC2A7*, glucose transporter 7; *SLC2A8*, glucose transporter 8; *FABP2*, fatty acid binding protein 2; *SLC16A10*, aromatic amino acid transporter; *SLC6A19*, neutral amino acid transporter; *SLC7A1*, cationic amino acid transporter; *SLC5A8*, sodium-coupled monocarboxylate transporter; *SLC16A1*, monocarboxylate transporter 1; *CCK*, cholecystokinin; *GLP2R*, glucagon-like peptide 2 receptor; *GCG*, glucagon; *SI*, sucrase isomaltase; *CNDP1*, carnosine dipeptidase; *NFKB1*, nuclear factor kappa B subunit 1; *CXCL8*, C-X-C motif chemokine ligand 8; *TGFB1*, transforming growth factor beta 1; *IFNG*, interferon gamma; *IL1A*, interleukin 1A; *TJP1*, tight junction protein 1; *OCLN*, occludin; *CLDN3*, claudin 3; *CLDN5*, claudin 5; *TLR2*, toll-like receptor 2; *TLR4*, toll-like receptor 4; *TLR5*, toll-like receptor 5; *MUC1*, mucin 1; *MUC2*, mucin 2; *ACTB*, actin beta; *B2M*, beta-2-microglobulin; *GAPDH*, glyceraldehyde-3-phosphate dehydrogenase; *PPIA*, peptidylprolyl isomerase A; *HPRT*, hypoxanthine phosphoribosyltransferase 1.

**Table 11:** Panel of genes analysed in the colon.

Group	Genes	Accession
Cytokines	*CXCL8*	NM_213867.1
	*IL1A*	NM_214029.1
	*IL1B*	NM_214055.1
	*IFNG*	NM_213948.1
Enzymes	*AOAH*	XM_021079244.1
	*CASP1*	NM_214162.1
	*PMRT5*	NM_001160093.1
	*TRAF2*	XM_005652719.1
	*TRAF3*	XM_005666443.2
	*TRAF6*	NM_001105286.1
Kinase	*CHUK*	NM_001114279.1
	*PRKAA1*	NM_001167633.1
	*MAPK1*	NM_001198922.1
	*MAP3K7*	NM_001114280.1
	*RIPK2*	XM_021089139.1
	*MTOR*	XM_003127584.6
	*SYK*	NM_001104952.1
	*JAK2*	NM_214113.1
Tight junctions	*CDH2*	XM_021096205.1
	*OCLN*	NM_001163647.2
	*TJP1*	XM_005659811.1
Mucins	*MUC1*	XM_021089728.1
	*MUC2*	XM_021082584.1
	*MUC4*	NM_001206344.2
Pathogen recognition receptors	*TLR1*	NM_001031775.1
	*TLR2*	NM_213761.1
	*TLR4*	NM_001113039.2
	*TLR6*	NM_213760.1
	*TLR7*	NM_001097434.1
	*TLR8*	NM_214187.1
	*MAVS*	NM_001097429.1
Ligand dependent nuclear receptor	*PPARG*	NM_214379.1
Suppresser of cytokine signalling	*SOCS1*	NM_001204768.1
	*SOCS3*	NM_001123196.1
Matrix metalloproteinases	*MMP2*	NM_214192.2
	*MMP3*	NM_001166308.1
	*MMP9*	NM_001038004.1
Transcription regulation	*JUN*	NM_213880.1
	*IRF3*	NM_213770.1
	*MYD88*	NM_001099923.1
	*NFKB1*	NM_001048232.1
	*PPARGC1A*	NM_213963.2
	*STAT2*	NM_213889.1
	*STAT3*	NM_001044580.1
	*TANK*	XM_003359533.4
	*TIRAP*	XM_003130060.4
	*TRAM1*	XM_001924618.6
Transmembrane receptor	*CD14*	NM_001097445.2
	*CLEC7A*	NM_001145866.1
	*DDX58*	NM_213804.2
	*TICAM1*	NM_001315738.1
Nutrient transporters	*SLC16A1*	NM_001128445.1
	*SLC16A7*	XM_003126337.5
Reference	*ACTB*	XM_003124280.
	*B2M*	NM_213978.1
	*GAPDH*	NM_001206359.1
	*G6PD*	XM_021080744.1
	*HPRT*	NM_001032376.2
	*PPIA*	NM_214353.1
	*RPL19*	XM_003131509.4
	*TBP*	XM_021085493.1

*CXCL8*, C–X–C motif chemokine ligand 8; *IL1A*, interleukin 1A; *IL1B*, interleukin 1B; *IFNG*, interferon gamma; *AOAH*, acyloxyacyl hydrolase; *CASP1*, caspase-1; *PRMT5*, protein arginine methyltransferase 5; *TRAF2*, TNF receptor associated factor 2; *TRAF3*, TNF receptor associated factor 3; *TRAF6*, TNF receptor associated factor 6; *CHUK*, component of inhibitor of nuclear factor kappa B kinase complex; *PRKAA1*, protein kinase AMP-activated catalytic subunit alpha 1; *MAPK1*, mitogen activate protein kinase 1; *MAP3K7*, mitogen-activated protein kinase kinase 7; *RIPK2*, receptor interacting serine/threonine kinase 2; *MTOR*, mechanistic target of rapamycin kinase; *SYK*, spleen associated tyrosine kinase; *JAK2*, janus kinase 2; *CDH2*, Cadherin 2; *OCLN*, occludin; *TJP1*, tight junction protein 1; *MUC1*, mucin 1; *MUC2*, mucin 2; *MUC4*, mucin 4; TLR1, toll-like receptor 1; *TLR2*, toll-like receptor 2; *TLR4*, toll-like receptor 4; *TLR6*, toll-like receptor 6; *TLR7*, toll-like receptor 7; *TLR8*, toll-like receptor 8; *MAVS*, mitochondrial antiviral signalling protein; *PPARG*, peroxisome proliferator activated receptor gamma; *SOCS1*, suppressor of cytokine signalling 1; *SOCS3*, suppressor of cytokine signalling 3; *MMP2*, matrix metalloproteinase 2; *MMP3*, matrix metalloproteinase 3; *MMP9*, matrix metalloproteinase 9; *JUN*, AP-1 transcription factor subunit; *IRF3*, interferon regulatory factor 3; *MYD88*, MYD88 innate immune signal transduction adaptor; *NFKB1*, nuclear factor kappa B subunit 1; *PPARGC1A*, PPARG coactivator 1 alpha; *STAT2*, signal transducer and activator of transcription 2; *STAT3*, signal transducer and activator of transcription 3; *TANK*, TRAF family member associated NFKB activator; *TIRAP*, TIR domain containing adaptor protein; *TRAM1*, translocation associated membrane protein 1; *CD14*, CD14 molecule; *CLEC7A*, C-type lectin domain containing 7A; *DDX58*, DExD/H-box helicase 58; *TICAM1*, toll like receptor adaptor molecule 1; *SLC16A1*, monocarboxylate transporter 1; *SLC16A7*, monocarboxylate transporter 7; *ACTB*, actin beta; *B2M*, beta-2-microglobulin; *GAPDH*, glyceraldehyde-3-phosphate dehydrogenase; *G6PD*, glucose-6-phosphate dehydrogenase; *HPRT*, hypoxanthine phosphoribosyltransferase 1; *PPIA*, peptidylprolyl isomerase A, *RPL19*, ribosomal protein L19; *TBP*, TATA-box binding protein.

**Table 12:** Oligonucleotide sequences of forward and reverse primers used for QPCR of bacterial 16 s rRNA.

Target Bacteria	Forward Primer (5′–3′) Reverse Primer (5′–3′)	Tm	Amplicon Size (bp)
Total bacteria	F: GTGCCAGCMGCCGCGGTAA	64.2	291
	R: GACTACCAGGGTATCTAAT	52.4	
*Enterobacteriaceae*	F: ATGGCTGTCGTCAGCTCGT	58.8	385
	R: CCTACTTCTTTTGCAACCCACTC	60.6	
*Lactobacillus* spp.	F: GAGGCAGCAGTAGGGAATCTTC	60.5	206
	R: CCAGCGTTGCCACCTACGTA	62.5	
*Bifidobacterium* spp.	F: CGCGTCYGGTGTGAAAG	62.5	244
	R: CCCCACATCCAGCATCCA	59.0	

Tm, melting temperature; bp, base pair.

## Supplementary Materials

The following are available online at https://www.mdpi.com/1660-3397/17/12/680/s1, Supplementary document 1: Figure S1. The effect of fucoidan on alpha diversity measures; Tables S1–S4: The effect of fucoidan on gene expression in the small and large intestine. Supplementary Table S1: Effect of fucoidan on the relative abundance of bacteria in the caecum. Supplementary Table S2: Effect of fucoidan on the relative abundance of bacteria in the colon.
