# Supplementary material for: The Effect of Increasing Inclusion Levels of a Fucoidan-Rich Extract Derived from Ascophyllum nodosum on Growth Performance and Aspects of Intestinal Health of Pigs Post-Weaning
Source: Mar Drugs. 2019 Nov 30;17(12):680. doi: 10.3390/md17120680 (PMC6950662; doi:10.3390/md17120680)
Supplement: Supplementary file 1 [file marinedrugs-17-00680-s001.pdf]

Supplementary

The Effect of Increasing Inclusion Levels of a Fucoidan Rich Extract Derived from Ascophyllum nodosum on Growth Performance and Aspects of Intestinal Health of Pigs Post-Weaning

Ruth Rattigan 1, Stafford Vigors 1, Gaurav Rajauria 1, Torres Sweeney 2, Kevin Thornton 2 and John V O'Doherty 1,*

Supplementary document 1. Alpha diversity & gene expression

Figure S1. The effect of fucoidan supplementation at 250ppm on Observed, Shannon and Simpson measures of alpha diversity in caecal and colonic digesta.

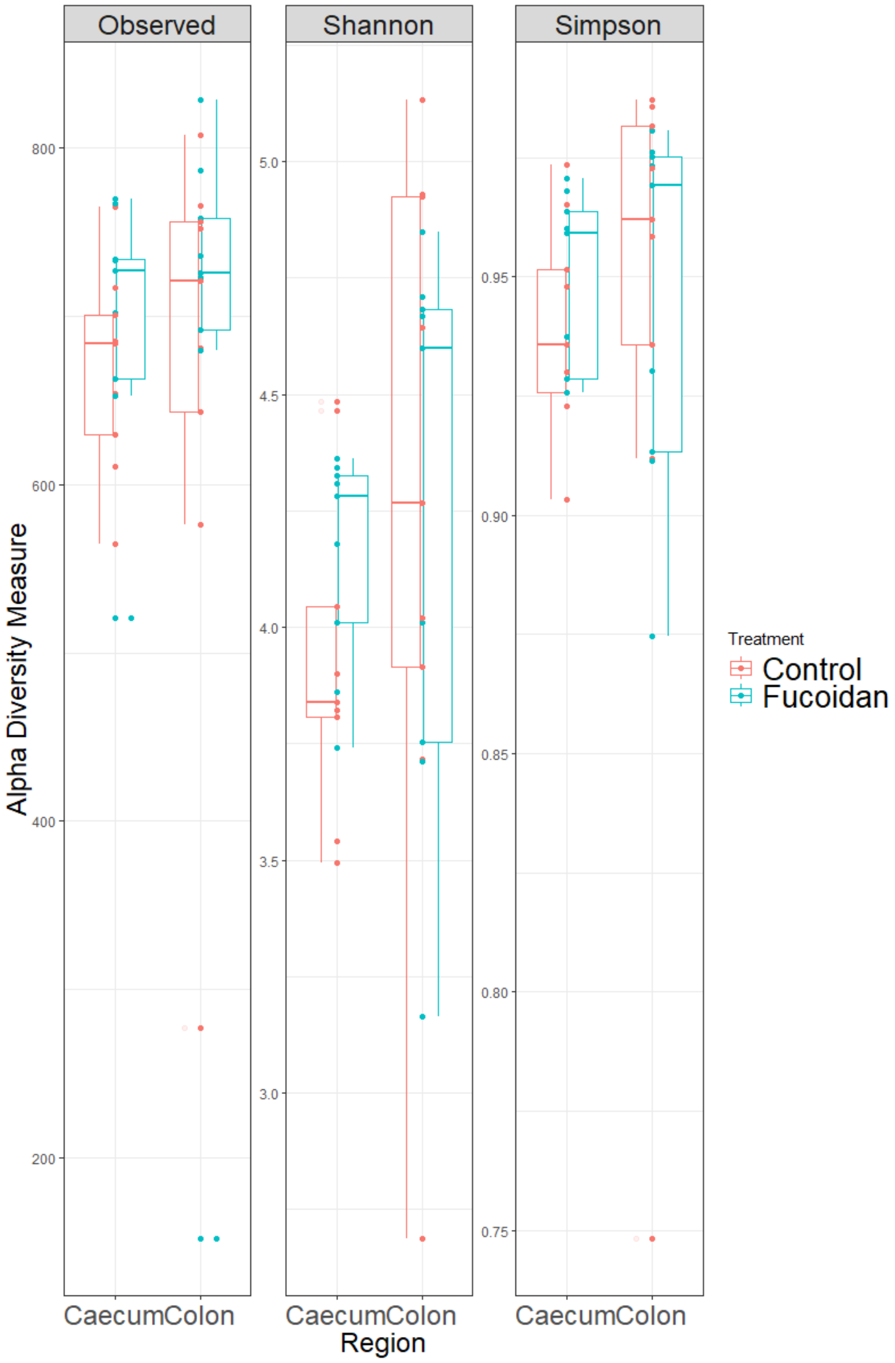

Table DS1. Effect of fucoidan on gene expression in the duodenum (Least-square means with their standard errors)

Group	Gene	Basal	Fucoidan 250ppm	SEM	P value
Digestive enzymes	*SI*	23195.53	21920.53	3734.92	0.813
	CNDP1	220.76	195.56	57.42	0.761
Nutrient transporters	*FABP2*	64101.33	63277.94	13214.97	0.966
	SLC2A1	340.44	103.05	47.20	0.364
	SLC2A2	3501.49	3760.72	603.38	0.766
	SLC2A5	979.44	789.62	90.78	0.163
	SLC2A7	508.99	496.96	88.62	0.925
	SLC2A8	226.76	401.46	66.18	0.083
	SLC16A1	2246.59	2698.03	218.08	0.165
	SLC15A1	3936.03	4139.35	585.09	0.810
	SLC5A1	11917.91	11381.36	1652.19	0.822
	SLC16A10	649.47	581.32	71.64	0.512
	SLC6A19	2623.95	2733.15	213.52	0.723
	SLC7A1	157.24	181.69	12.93	0.202
	SLC5A8	3214.00	4059.56	199.78	0.010
Appetite regulators	*GLP2R*	153.47	163.79	10.00	0.479
	GCG	723.21	264.69	275.57	0.261
	CCK	825.90	813.21	98.49	0.929
Tight junctions	*CLDN3*	2284.64	2545.28	135.22	0.194
	CLDN5	65.53	55.71	3.18	0.047
	OCLN	2290.33	2235.59	148.52	0.798
	TJP1	1050.29	1051.15	28.50	0.983
Inflammatory markers	*NFKB1*	561.69	598.47	16.04	0.127
	IFNG	90.61	110.40	16.36	0.408
	IL1A	68.22	109.07	20.33	0.177
	CXCL8	1767.21	2244.65	265.10	0.227
	TGFB1	119.51	112.87	7.79	0.557
Mucins	*MUC1*	47.61	51.23	7.61	0.742
	MUC2	6862.70	7717.75	786.46	0.455
Toll-like receptors	*TLR2*	141.18	177.63	30.55	0.413
	TLR4	181.46	208.51	18.96	0.330
	TLR5	112.00	94.07	12.99	0.347

SI, sucrase isomaltase; *CNDP1*, carnosine dipeptidase 1; *FABP2*, fatty acid binding protein 2; *SLC2A1*, glucose transporter 1; *SLC2A2*, glucose transporter 2, *SLC2A5*, glucose transporter 5; *SLC2A7*, glucose transporter 7, *SLC2A8*, glucose transporter 8; *SLC16A1*, monocarboxylate transporter 1; *SLC15A1*, peptide transporter 1; *SLC5A1*, sodium glucose co-transporter; *SLC16A10*, aromatic amino acid transporter; *SLC6A19*, neutral amino acid transporter; *SLC7A1*, cationic amino acid transporter; *SLC5A8*, sodium-coupled monocarboxylate transporter; *GLP2R*, glucagon-like peptide 2 receptor; *GCG*, glucagon; *CCK*, cholecystokinin; *CLDN3*, claudin 3; *CLDN5*, claudin 5; *OCLN*, occludin; *TJP1*, tight junction protein 1; *NFKB1*, nuclear factor kappa B subunit 1; *IFNG*, interferon gamma; *IL1A*, interleukin 1A; *CXCL8*, C-X-C motif chemokine ligand 8; *TGFB1*, transforming growth factor beta 1; *MUC1*, mucin 1; *MUC2*, mucin 2; *TLR2*, toll-like receptor 2; *TLR4*, toll-like receptor 4; *TLR5*, toll-like receptor 5

Table DS2. Effect of fucoidan on gene expression in the jejunum (Least-square means with their standard errors)

Group	Gene	Basal	Fucoidan 250ppm	SEM	P value
Digestive enzymes	*SI*	20997.41	8785.56	3261.91	0.020
	CNDP1	191.24	158.03	48.28	0.636
Nutrient transporters	*FABP2*	11759.37	7874.56	2276.07	0.248
	SLC2A1	200.39	228.18	17.56	0.282
	SLC2A2	1153.30	966.55	342.81	0.706
	SLC2A5	268.33	143.01	54.12	0.126
	SLC2A7	136.43	98.44	39.45	0.507
	SLC2A8	56.93	54.83	3.80	0.701
	SLC16A1	1131.83	1096.72	70.53	0.730
	SLC15A1	1218.79	648.81	190.18	0.054
	SLC5A1	7466.46	2564.99	937.75	0.003
	SLC16A10	642.93	507.07	103.11	0.367
	SLC6A19	1441.14	1256.39	339.83	0.706
	SLC7A1	409.44	548.21	48.40	0.062
	SLC5A8	2041.92	1704.22	515.60	0.650
Appetite regulators	*GLP2R*	81.51	59.61	16.58	0.366
	GCG	2590.99	2093.25	674.91	0.610
	CCK	148.38	204.13	21.25	0.085
Tight junctions	*CLDN3*	803.60	969.62	211.43	0.588
	CLDN5	52.29	54.58	4.15	0.703
	OCLN	1217.85	966.23	327.44	0.595
	TJP1	1490.73	1346.44	74.08	0.190
Inflammatory markers	*NFKB1*	865.45	777.13	41.40	0.154
	IFNG	109.66	78.75	15.64	0.184
	IL1A	70.48	58.60	7.82	0.300
	CXCL8	914.94	890.35	206.24	0.934
	TGFB1	178.64	176.55	8.80	0.869
Mucins	*MUC1*	29.10	23.52	6.07	0.526
	MUC2	4748.24	5777.23	505.24	0.174
Toll-like receptors	*TLR2*	769.85	679.54	150.24	0.677
	TLR4	198.35	155.29	23.22	0.211
	TLR5	99.51	73.03	25.47	0.474

SI, sucrase isomaltase; *CNDP1*, carnosine dipeptidase 1; *FABP2*, fatty acid binding protein 2; *SLC2A1*, glucose transporter 1; *SLC2A2*, glucose transporter 2, *SLC2A5*, glucose transporter 5; *SLC2A7*, glucose transporter 7, *SLC2A8*, glucose transporter 8; *SLC16A1*, monocarboxylate transporter 1; *SLC15A1*, peptide transporter 1; *SLC5A1*, sodium glucose co-transporter; *SLC16A10*, aromatic amino acid transporter; *SLC6A19*, neutral amino acid transporter 19; *SLC7A1*, cationic amino acid transporter; *SLC5A8*, sodium-coupled monocarboxylate transporter; *GLP2R*, glucagon-like peptide 2 receptor; *GCG*, glucagon; *CCK*, cholecystokinin; *CLDN3*, claudin 3; *CLDN5*, claudin 5; *OCLN*, occludin; *TJP1*, tight junction protein 1; *NFKB1*, nuclear factor kappa B subunit 1; *IFNG*, interferon gamma; *IL1A*, interleukin 1A; *CXCL8*, C-X-C motif chemokine ligand 8; *TGFB1*, transforming growth factor beta 1; mucin 1; *MUC2*, mucin 2; *TLR2*, toll-like receptor 2; *TLR4*, toll-like receptor 4; *TLR5*, toll-like receptor 5

Table DS3. Effect of fucoidan on gene expression in the ileum (Least-square means with their standard errors)

Group	Gene	Basal	Fucoidan 250ppm	SEM	P value
Digestive enzymes	*SI*	40234.54	28707.41	4811.98	0.110
	CNDP1	172.39	140.36	21.54	0.309
Nutrient transporters	*FABP2*	13068.53	8027.51	1440.41	0.025
	SLC2A1	149.51	165.37	13.95	0.433
	SLC2A2	1872.01	1575.46	254.39	0.422
	SLC2A5	506.90	403.25	68.63	0.301
	SLC2A7	204.19	174.82	32.05	0.526
	SLC2A8	25.11	31.34	2.30	0.073
	SLC16A1	1021.74	1023.06	60.31	0.988
	SLC15A1	2294.29	1786.68	265.32	0.195
	SLC5A1	14956.02	9008.17	1924.65	0.044
	SLC16A10	786.41	639.06	93.35	0.281
	SLC6A19	2650.79	1948.00	258.98	0.073
	SLC7A1	89.14	142.59	24.61	0.144
	SLC5A8	2633.70	2373.21	289.25	0.533
Appetite regulators	*GCG*	4187.67	3642.47	464.53	0.419
	GLP2R	101.05	84.96	11.59	0.341
	CCK	24.06	43.00	7.99	0.113
Tight junctions	*CLDN3*	1899.47	1651.94	216.89	0.432
	CLDN5	66.49	54.56	5.43	0.140
	OCLN	1851.99	1436.42	167.45	0.098
	TJP1	1159.50	987.10	55.73	0.044
Inflammatory markers	*NFKB1*	544.31	556.49	23.51	0.719
	IFNG	140.51	115.90	16.17	0.298
	IL1A	48.17	42.39	5.43	0.462
	CXCL8	1161.11	1075.58	143.69	0.679
	TGFB1	83.44	102.02	10.18	0.215
Mucins	*MUC1*	41.99	37.04	6.77	0.612
	MUC2	8246.34	8618.90	806.64	0.748
Toll-like receptors	*TLR2*	938.68	1002.12	139.22	0.752
	TLR4	134.28	140.12	14.49	0.779
	TLR5	144.46	110.97	18.41	0.217

SI, sucrase isomaltase; *CNDP1*, carnosine dipeptidase 1; *FABP2*, fatty acid binding protein 2; *SLC2A1*, glucose transporter 1; *SLC2A2*, glucose transporter 2, *SLC2A5*, glucose transporter 5; *SLC2A7*, glucose transporter 7, *SLC2A8*, glucose transporter 8; *SLC16A1*, monocarboxylate transporter 1; *SLC15A1*, peptide transporter 1; *SLC5A1*, sodium glucose co-transporter; *SLC16A10*, aromatic amino acid transporter; *SLC6A19*, neutral amino acid transporter 19; *SLC7A1*, cationic amino acid transporter; *SLC5A8*, sodium-coupled monocarboxylate transporter; *GLP2R*, glucagon-like peptide 2 receptor; *GCG*, glucagon; *CCK*, cholecystokinin; *CLDN3*, claudin 3; *CLDN5*, claudin 5; *OCLN*, occludin; *TJP1*, tight junction protein 1; *NFKB1*, nuclear factor kappa B subunit 1; *IFNG*, interferon gamma; *IL1A*, interleukin 1A; *CXCL8*, C-X-C motif chemokine ligand 8; *TGFB1*, transforming growth factor beta 1; *MUC1*, mucin 1; *MUC2*, mucin 2; *TLR2*, toll-like receptor 2; *TLR4*, toll-like receptor 4; *TLR5*, toll-like receptor 5

Table DS4. Effect of fucoidan on gene expression in the colon (Least-square means with their standard errors)

Group	Gene	Basal	Fucoidan 250ppm	SEM	P Value
Enzymes	*AOAH*	160.72	149.84	15.72	0.632
	CASP1	1458.35	936.51	280.61	0.209
	PMRT5	515.30	512.51	19.68	0.922
	TRAF2	70.86	72.59	3.08	0.698
	TRAF3	152.82	130.90	7.12	0.047
	TRAF6	417.17	413.92	20.81	0.914
Tight junctions	*CDH2*	74.97	80.05	12.52	0.778
	OCLN	1272.24	1171.69	87.19	0.428
	TJP1	622.59	617.08	22.25	0.863
Cytokines	*IL1A*	303.97	328.09	36.94	0.651
	IL1B	90.05	142.08	20.41	0.092
	IFNG	60.87	54.94	9.43	0.663
	CXCL8	823.45	706.52	91.54	0.381
Transcription regulators	*IRF3*	920.89	767.48	82.10	0.206
	MYD88	543.80	562.13	21.20	0.550
	NFKB1	317.40	294.78	14.36	0.283
	PPARGC1A	346.96	362.88	43.06	0.797
	STAT2	1562.74	1372.98	94.69	0.177
	STAT3	1303.63	1285.62	66.04	0.850
	JUN	1405.02	1399.53	121.26	0.975
	TIRAP	55.71	54.68	3.42	0.835
	TANK	483.14	439.34	19.27	0.129
	TRAM1	5043.20	5049.60	212.22	0.983
Kinases	*JAK2*	453.33	434.17	20.57	0.520
	MAPK1	1687.52	1827.27	86.05	0.269
	RIPK2	173.70	172.15	6.30	0.864
	MTOR	233.62	239.30	7.14	0.582
	SYK	334.29	274.08	36.96	0.268
	CHUK	437.50	426.45	10.68	0.476
	PRKAA1	849.68	832.03	26.85	0.649
	MAP3K7	843.38	828.61	23.36	0.661
Matrix metalloproteinases	*MMP2*	550.04	431.50	41.81	0.064
	MMP3	345.99	311.68	90.01	0.791
	MMP9	68.37	75.37	23.35	0.835
Mucins	*MUC1*	1246.01	1267.13	170.55	0.931
	MUC2	5322.72	5344.22	551.98	0.978
	MUC4	297.90	302.52	43.65	0.941
Ligand dependent	*PPARG*	1398.30	1640.02	121.04	0.179
	TLR1	261.14	227.11	21.61	0.283
	TLR2	755.79	910.52	97.41	0.279
	TLR4	231.74	267.22	17.56	0.174
	TLR6	247.34	240.93	21.80	0.838
	TLR7	186.82	153.59	16.08	0.165
	TLR8	209.45	209.00	28.49	0.991
	MAVS	442.66	389.90	24.69	0.152

Suppressor of cytokine signalling	*SOCS1*	327.43	336.30	42.10	0.884
	SOCS3	670.32	777.06	78.27	0.350
Transmembrane receptor	*TICAM*	251.13	238.27	15.11	0.556
	DDX58	2789.66	1829.72	232.24	0.011
	CD14	45.66	48.68	5.01	0.676
	CLEC7A	99.98	67.56	19.31	0.254
Nutrient transporters	*SLC16A1*	4859.63	6303.03	728.97	0.182
	SLC16A7	210.16	206.66	9.97	0.807

AOAH, acyloxyacyl hydrolase; *CASP1*, caspase-1; *PRMT5,* protein arginine methyltransferase 5; *TRAF2*, TNF receptor associated factor 2; *TRAF3,* TNF receptor associated factor 3; *TRAF6*, TNF receptor associated factor 6; *CDH2*, cadherin 2; *OCLN*, occludin; *TJP1*, tight junction protein 1; *IL1A*, interleukin 1A; *IL1B*, interleukin 1B; *IFNG*, interferon gamma; *CXCL8*, C-X-C motif chemokine ligand 8; *IRF3*, interferon regulatory factor 3; *MYD88*, MYD88 innate immune signal transduction adaptor; *NFKB1*, nuclear factor kappa B subunit 1; *PPARGC1A*, PPARG coactivator 1 alpha; *STAT2*, signal transducer and activator of transcription 2; *STAT3*, signal transducer and activator of transcription 3; *JUN,* AP-1 transcription factor subunit; *TIRAP*, TIR domain containing adaptor protein; *TANK*, TRAF family member associated NFKB activator; *TRAM1*, translocation associated membrane protein 1; *JAK2,* janus kinase 2; *MAPK1*, mitogen activate protein kinase 1; *RIPK2*, receptor interacting serine/threonine kinase 2; *MTOR*, mechanistic target of rapamycin kinase; *SYK*, spleen associated tyrosine kinase; *CHUK*, component of inhibitor of nuclear factor kappa B kinase complex; *PRKAA1*, protein kinase AMP-activated catalytic subunit alpha 1; *MAP3K7*, mitogen-activated protein kinase kinase kinase 7; *MMP2*, matrix metalloproteinase 2; *MMP3*, matrix metalloproteinase 3; *MMP9*, matrix metalloproteinase 9; *MUC1*, mucin 1; *MUC2*, mucin 2; *MUC4*, mucin 4; *PPARG*, peroxisome proliferator activated receptor gamma; *TLR1*, toll-like receptor 1; *TLR2*, toll-like receptor 2; *TLR4,* toll-like receptor 4; *TLR6,* toll-like receptor 6; *TLR7*, toll-like receptor7; *TLR8,* toll-like receptor 8; *MAVS*, mitochondrial antiviral signalling protein; *SOCS1,* suppressor of cytokine signalling 1; *SOCS3*, suppressor of cytokine signalling 3; *TICAM1,* toll like receptor adaptor molecule 1; *DDX58*, DExD/H-box helicase 58; *CD14*, CD14 molecule; *CLEC7A*, C-type lectin domain containing 7A; *SLC16A1*, monocarboxylate transporter 1; *SLC16A7*, monocarboxylate transporter 7

Table S1. The effect of fucoidan on the relative abundance of bacteria in the caecal digesta

Basal	Fucoidan	SEM	P value	OTU id	Phylum	Class	Order	Family	Genus	Species
**Phylum**										
27.585	26.872	2.026	0.246	New.CleanUp.ReferenceOTU122441	Firmicutes					
0.069	0.084	0.013	0.859	363794	Actinobacteria					
0.040	0.039	0.014	0.859	New.ReferenceOTU3654	Fibrobacteres					
1.534	1.428	0.493	0.859	68837	Spirochaetes					
0.211	0.181	0.060	0.859	4374042	Deferribacteres					
19.437	16.709	2.551	0.246	113756	Proteobacteria					
50.824	54.225	2.277	0.277	568118	Bacteroidetes					
0.199	0.123	0.110	0.859	1654477	Fusobacteria					
0.100	0.339	0.073	0.358	185593	Tenericutes					
**Family**										
12.773	13.297	1.702	0.427	New.CleanUp.ReferenceOTU122441	Firmicutes	Clostridia	Clostridiales	Lachnospiraceae		
0.020	0.040	0.012	0.924	644244	Firmicutes	Clostridia	Clostridiales	Christensenellaceae		
0.075	0.091	0.014	0.713	363794	Actinobacteria	Coriobacteriia	Coriobacteriales	Coriobacteriaceae		
0.044	0.043	0.015	0.573	New.ReferenceOTU3654	Fibrobacteres	Fibrobacteria	Fibrobacterales	Fibrobacteraceae		
1.677	1.588	0.540	0.481	68837	Spirochaetes	Spirochaetes	Spirochaetales	Spirochaetaceae		
1.588	1.089	0.659	0.357	163857	Proteobacteria	Gammaproteobacteria	Aeromonadales	Succinivibrionaceae		
1.400	0.612	0.538	0.818	359779	Proteobacteria	Gammaproteobacteria	Pasteurellales	Pasteurellaceae		
4.846	2.398	1.285	0.346	1111294	Proteobacteria	Gammaproteobacteria	Enterobacteriales	Enterobacteriaceae		
0.152	0.233	0.034	0.530	333380	Proteobacteria	Betaproteobacteria	Burkholderiales	Alcaligenaceae		
0.002	0.007	0.002	0.530	360508	Proteobacteria	Betaproteobacteria	Burkholderiales	Oxalobacteraceae		
0.227	0.194	0.064	0.665	4374042	Deferribacteres	Deferribacteres	Deferribacterales	Deferribacteraceae		
0.097	0.476	0.108	0.155	30569	Proteobacteria	Deltaproteobacteria	Desulfovibrionales	Desulfovibrionaceae		
11.386	11.327	1.758	0.481	113756	Proteobacteria	Epsilonproteobacteria	Campylobacterales	Campylobacteraceae		
0.171	1.154	0.295	0.182	311173	Proteobacteria	Epsilonproteobacteria	Campylobacterales	Helicobacteraceae		
1.510	0.947	0.717	0.530	New.ReferenceOTU2302	Bacteroidetes	Bacteroidia	Bacteroidales	Bacteroidaceae		
35.897	36.890	3.195	0.362	568118	Bacteroidetes	Bacteroidia	Bacteroidales	Prevotellaceae		
11.795	12.653	1.295	0.345	20534	Bacteroidetes	Bacteroidia	Bacteroidales	[Paraprevotellaceae]		
0.000	0.002	0.001	0.357	4307122	Bacteroidetes	Bacteroidia	Bacteroidales	[Odoribacteraceae]		
0.633	1.217	0.261	0.701	28974	Bacteroidetes	Bacteroidia	Bacteroidales	Porphyromonadaceae		
1.156	1.393	0.170	0.426	577228	Bacteroidetes	Bacteroidia	Bacteroidales	S24-7		
0.040	0.006	0.015	0.519	New.ReferenceOTU2170	Bacteroidetes	Bacteroidia	Bacteroidales	BS11		
0.417	1.028	0.372	0.008	New.ReferenceOTU3588	Bacteroidetes	Bacteroidia	Bacteroidales	RF16		
0.067	0.137	0.038	0.887	16915	Bacteroidetes	Bacteroidia	Bacteroidales	p-2534-18B5		
0.000	0.054	0.026		1143674	Tenericutes	Mollicutes	Mycoplasmatales	Mycoplasmataceae		
0.235	0.148	0.131	0.782	1654477	Fusobacteria	Fusobacteriia	Fusobacteriales	Fusobacteriaceae		
0.107	0.292	0.064	0.678	185593	Tenericutes	Mollicutes	Anaeroplasmatales	Anaeroplasmataceae		
0.189	0.004	0.076	0.053	368490	Firmicutes	Bacilli	Turicibacterales	Turicibacteraceae		
0.062	0.013	0.021	0.345	349024	Firmicutes	Bacilli	Lactobacillales	Streptococcaceae		
3.105	2.214	0.898	0.427	302975	Firmicutes	Bacilli	Lactobacillales	Lactobacillaceae		
2.203	2.260	0.579	0.481	New.ReferenceOTU1058	Firmicutes	Clostridia	Clostridiales	Veillonellaceae		
0.728	0.539	0.154	0.346	215963	Firmicutes	Clostridia	Clostridiales	Clostridiaceae		
0.016	0.020	0.007	0.481	New.ReferenceOTU2707	Firmicutes	Clostridia	Clostridiales	Peptococcaceae		
0.017	0.004	0.005	0.155	712677	Firmicutes	Clostridia	Clostridiales	Peptostreptococcaceae		
0.093	0.094	0.023	0.481	302433	Firmicutes	Clostridia	Clostridiales	[Mogibacteriaceae]		
7.270	7.537	0.739	0.364	851865	Firmicutes	Clostridia	Clostridiales	Ruminococcaceae		
**Genus**										
5.232	5.257	1.384	0.985	New.CleanUp.ReferenceOTU122441	Firmicutes	Clostridia	Clostridiales	Lachnospiraceae	Roseburia	
0.939	0.590	0.202	0.614	843553	Firmicutes	Clostridia	Clostridiales	Lachnospiraceae	Lachnospira	
0.685	0.581	0.134	0.708	1107057	Firmicutes	Clostridia	Clostridiales	Lachnospiraceae	Coprococcus	
0.080	0.027	0.025	0.797	4364564	Firmicutes	Clostridia	Clostridiales	Lachnospiraceae	Butyrivibrio	
0.087	0.129	0.017	0.797	1076587	Firmicutes	Clostridia	Clostridiales	Lachnospiraceae	Dorea	
0.123	0.109	0.022	0.911	696563	Firmicutes	Clostridia	Clostridiales	Lachnospiraceae	Blautia	
0.032	0.005	0.014	0.911	New.ReferenceOTU2736	Firmicutes	Clostridia	Clostridiales	Lachnospiraceae	Epulopiscium	
0.003	0.015	0.005	0.614	367139	Actinobacteria	Coriobacteriia	Coriobacteriales	Coriobacteriaceae	Slackia	
0.030	0.062	0.013	0.614	363794	Actinobacteria	Coriobacteriia	Coriobacteriales	Coriobacteriaceae	Collinsella	
0.053	0.052	0.018	0.911	New.ReferenceOTU3654	Fibrobacteres	Fibrobacteria	Fibrobacterales	Fibrobacteraceae	Fibrobacter	
1.985	1.927	0.628	0.901	68837	Spirochaetes	Spirochaetes	Spirochaetales	Spirochaetaceae	Treponema	
0.029	0.397	0.100	0.183	587570	Proteobacteria	Gammaproteobacteria	Aeromonadales	Succinivibrionaceae	Anaerobiospirillum	
1.802	0.891	0.758	0.183	163857	Proteobacteria	Gammaproteobacteria	Aeromonadales	Succinivibrionaceae	Succinivibrio	
0.057	0.003	0.027	0.797	9498	Proteobacteria	Gammaproteobacteria	Pasteurellales	Pasteurellaceae	Aggregatibacter	
1.504	0.730	0.570	0.614	359779	Proteobacteria	Gammaproteobacteria	Pasteurellales	Pasteurellaceae	Actinobacillus	
0.182	0.294	0.045	0.911	333380	Proteobacteria	Betaproteobacteria	Burkholderiales	Alcaligenaceae	Sutterella	
0.002	0.009	0.003	0.797	360508	Proteobacteria	Betaproteobacteria	Burkholderiales	Oxalobacteraceae	Oxalobacter	
0.269	0.227	0.073	0.911	4374042	Deferribacteres	Deferribacteres	Deferribacterales	Deferribacteraceae	Mucispirillum	
0.082	0.480	0.114	0.183	30569	Proteobacteria	Deltaproteobacteria	Desulfovibrionales	Desulfovibrionaceae	Desulfovibrio	
0.001	0.013	0.004	0.481	New.ReferenceOTU2103	Proteobacteria	Deltaproteobacteria	Desulfovibrionales	Desulfovibrionaceae	Bilophila	
14.017	13.943	2.205	0.888	113756	Proteobacteria	Epsilonproteobacteria	Campylobacterales	Campylobacteraceae	Campylobacter	
0.067	0.804	0.181	0.002	311173	Proteobacteria	Epsilonproteobacteria	Campylobacterales	Helicobacteraceae	Helicobacter	
1.950	1.335	0.959	0.614	New.ReferenceOTU2302	Bacteroidetes	Bacteroidia	Bacteroidales	Bacteroidaceae	Bacteroides	
42.733	43.908	3.493	0.911	568118	Bacteroidetes	Bacteroidia	Bacteroidales	Prevotellaceae	Prevotella	
0.366	0.547	0.138	0.968	4435235	Bacteroidetes	Bacteroidia	Bacteroidales	[Paraprevotellaceae]	YRC22	
11.977	11.917	1.562	0.797	20534	Bacteroidetes	Bacteroidia	Bacteroidales	[Paraprevotellaceae]	[Prevotella]	
1.889	2.996	0.409	0.911	300853	Bacteroidetes	Bacteroidia	Bacteroidales	[Paraprevotellaceae]	CF231	
0.000	0.003	0.001	0.479	4307122	Bacteroidetes	Bacteroidia	Bacteroidales	[Odoribacteraceae]	Odoribacter	
0.793	1.583	0.366	0.911	28974	Bacteroidetes	Bacteroidia	Bacteroidales	Porphyromonadaceae	Parabacteroides	
0.002	0.000	0.000	0.479	New.ReferenceOTU1695	Bacteroidetes	Bacteroidia	Bacteroidales	Porphyromonadaceae	Paludibacter	
0.000	0.067	0.033		1143674	Tenericutes	Mollicutes	Mycoplasmatales	Mycoplasmataceae	Mycoplasma	
0.306	0.226	0.179	0.797	1654477	Fusobacteria	Fusobacteriia	Fusobacteriales	Fusobacteriaceae	Fusobacterium	
0.041	0.216	0.073	0.911	New.ReferenceOTU3606	Tenericutes	Mollicutes	Anaeroplasmatales	Anaeroplasmataceae	Anaeroplasma	
0.211	0.005	0.084	0.002	368490	Firmicutes	Bacilli	Turicibacterales	Turicibacteraceae	Turicibacter	
0.070	0.015	0.023	0.507	349024	Firmicutes	Bacilli	Lactobacillales	Streptococcaceae	Streptococcus	
3.706	2.569	1.074	0.797	302975	Firmicutes	Bacilli	Lactobacillales	Lactobacillaceae	Lactobacillus	
0.002	0.013	0.004	0.593	25947	Firmicutes	Clostridia	Clostridiales	Veillonellaceae	Acidaminococcus	
0.045	0.052	0.007	0.955	916143	Firmicutes	Clostridia	Clostridiales	Veillonellaceae	Phascolarctobacterium	
0.083	0.058	0.025	0.911	149335	Firmicutes	Clostridia	Clostridiales	Veillonellaceae	Mitsuokella	
2.439	2.435	0.690	0.888	New.ReferenceOTU1058	Firmicutes	Clostridia	Clostridiales	Veillonellaceae	Anaerovibrio	
0.053	0.061	0.019	0.985	266210	Firmicutes	Clostridia	Clostridiales	Veillonellaceae	Megasphaera	
0.000	0.038	0.013	0.221	264552	Firmicutes	Clostridia	Clostridiales	Veillonellaceae	Dialister	
0.419	0.318	0.102	0.880	215963	Firmicutes	Clostridia	Clostridiales	Clostridiaceae	Clostridium	
0.020	0.029	0.011	0.911	New.ReferenceOTU2707	Firmicutes	Clostridia	Clostridiales	Peptococcaceae	rc4-4	
0.054	0.010	0.022	0.888	1112364	Firmicutes	Clostridia	Clostridiales	[Mogibacteriaceae]	Anaerovorax	
3.303	2.410	0.794	0.911	851865	Firmicutes	Clostridia	Clostridiales	Ruminococcaceae	Faecalibacterium	
0.349	0.347	0.062	0.888	148925	Firmicutes	Clostridia	Clostridiales	Ruminococcaceae	Ruminococcus	
1.871	2.288	0.207	0.955	310886	Firmicutes	Clostridia	Clostridiales	Ruminococcaceae	Oscillospira	
0.045	0.002	0.019	0.614	1111191	Firmicutes	Clostridia	Clostridiales	Lachnospiraceae	[Ruminococcus]	
0.014	0.007	0.002	0.708	New.ReferenceOTU1761	Firmicutes	Clostridia	Clostridiales	Lachnospiraceae	Anaerostipes	
**Species**										
0.016	0.003	0.004	0.829	362501	Firmicutes	Clostridia	Clostridiales	Lachnospiraceae	Coprococcus	eutactus
0.092	0.192	0.052	0.829	1076587	Firmicutes	Clostridia	Clostridiales	Lachnospiraceae	Dorea	formicigenerans
0.074	0.158	0.033	0.646	363794	Actinobacteria	Coriobacteriia	Coriobacteriales	Coriobacteriaceae	Collinsella	aerofaciens
0.022	0.014	0.011	0.829	638485	Fibrobacteres	Fibrobacteria	Fibrobacterales	Fibrobacteraceae	Fibrobacter	succinogenes
0.013	0.595	0.279	0.923	360508	Proteobacteria	Betaproteobacteria	Burkholderiales	Oxalobacteraceae	Oxalobacter	formigenes
0.785	0.718	0.216	0.923	4374042	Deferribacteres	Deferribacteres	Deferribacterales	Deferribacteraceae	Mucispirillum	schaedleri
0.178	0.377	0.182	0.829	365496	Bacteroidetes	Bacteroidia	Bacteroidales	Bacteroidaceae	Bacteroides	plebeius
21.987	24.578	2.835	0.923	591785	Bacteroidetes	Bacteroidia	Bacteroidales	Prevotellaceae	Prevotella	stercorea
64.518	61.626	4.948	0.923	568118	Bacteroidetes	Bacteroidia	Bacteroidales	Prevotellaceae	Prevotella	copri
0.127	0.037	0.053	0.829	349024	Firmicutes	Bacilli	Lactobacillales	Streptococcaceae	Streptococcus	luteciae
0.048	0.018	0.022	0.931	New.ReferenceOTU3048	Firmicutes	Bacilli	Lactobacillales	Lactobacillaceae	Lactobacillus	mucosae
0.170	0.150	0.056	0.923	149335	Firmicutes	Clostridia	Clostridiales	Veillonellaceae	Mitsuokella	multacida
11.538	9.721	2.580	0.923	851865	Firmicutes	Clostridia	Clostridiales	Ruminococcaceae	Faecalibacterium	prausnitzii
0.163	1.576	0.722	0.829	369602	Firmicutes	Clostridia	Clostridiales	Ruminococcaceae	Ruminococcus	bromii
0.092	0.001	0.041	0.923	New.ReferenceOTU3836	Firmicutes	Clostridia	Clostridiales	Ruminococcaceae	Ruminococcus	flavefaciens
0.109	0.005	0.044	0.646	1111191	Firmicutes	Clostridia	Clostridiales	Lachnospiraceae	[Ruminococcus]	gnavus
0.069	0.231	0.082	0.923	New.CleanUp.ReferenceOTU17239	Firmicutes	Clostridia	Clostridiales	Lachnospiraceae	Roseburia	faecis

Table S2. Effect of fucoidan supplementation on relative bacterial abundance in the colonic digesta

Basal	Fucoidan	SEM	P value	OUT ID	Phylum	Class	Order	Family	Genus	Species
**Phylum**										
29.571	28.765	2.822	0.996	New.CleanUp.ReferenceOTU122441	Firmicutes					
0.087	0.090	0.024	0.996	288683	Actinobacteria					
0.093	0.096	0.035	0.996	New.ReferenceOTU3654	Fibrobacteres					
2.487	1.833	0.569	0.670	924224	Spirochaetes					
0.613	0.419	0.209	0.996	25453	Deferribacteres					
12.075	12.430	2.301	0.996	113756	Proteobacteria					
54.580	56.078	2.471	0.996	568118	Bacteroidetes					
0.314	0.002	0.153	0.670	1654477	Fusobacteria					
0.179	0.287	0.056	0.996	New.ReferenceOTU3606	Tenericutes					
**Family**										
13.396	12.999	1.165	0.790	New.CleanUp.ReferenceOTU122441	Firmicutes	Clostridia	Clostridiales	Lachnospiraceae		
1.521	0.665	0.538	0.105	4451049	Firmicutes	Clostridia	Clostridiales	Christensenellaceae		
0.102	0.101	0.028	0.819	288683	Actinobacteria	Coriobacteriia	Coriobacteriales	Coriobacteriaceae		
0.109	0.110	0.041	0.790	New.ReferenceOTU3654	Fibrobacteres	Fibrobacteria	Fibrobacterales	Fibrobacteraceae		
2.923	2.062	0.661	0.445	924224	Spirochaetes	Spirochaetes	Spirochaetales	Spirochaetaceae		
0.528	0.751	0.220	0.790	517804	Proteobacteria	Gammaproteobacteria	Aeromonadales	Succinivibrionaceae		
0.071	0.343	0.108	0.187	359779	Proteobacteria	Gammaproteobacteria	Pasteurellales	Pasteurellaceae		
3.317	1.852	0.894	0.790	1111294	Proteobacteria	Gammaproteobacteria	Enterobacteriales	Enterobacteriaceae		
0.142	0.303	0.062	0.350	333380	Proteobacteria	Betaproteobacteria	Burkholderiales	Alcaligenaceae		
0.014	0.010	0.004	0.744	360508	Proteobacteria	Betaproteobacteria	Burkholderiales	Oxalobacteraceae		
0.743	0.472	0.257	0.744	25453	Deferribacteres	Deferribacteres	Deferribacterales	Deferribacteraceae		
0.327	0.370	0.079	0.923	4453773	Proteobacteria	Deltaproteobacteria	Desulfovibrionales	Desulfovibrionaceae		
5.671	7.978	1.317	0.807	113756	Proteobacteria	Epsilonproteobacteria	Campylobacterales	Campylobacteraceae		
1.092	1.057	0.483	0.923	311173	Proteobacteria	Epsilonproteobacteria	Campylobacterales	Helicobacteraceae		
2.249	0.332	0.974	0.923	New.ReferenceOTU2302	Bacteroidetes	Bacteroidia	Bacteroidales	Bacteroidaceae		
35.042	36.265	3.541	0.807	568118	Bacteroidetes	Bacteroidia	Bacteroidales	Prevotellaceae		
12.458	14.969	1.538	0.790	20534	Bacteroidetes	Bacteroidia	Bacteroidales	[Paraprevotellaceae]		
0.000	0.002	0.001	0.199	4307122	Bacteroidetes	Bacteroidia	Bacteroidales	[Odoribacteraceae]		
1.079	0.932	0.175	0.744	921813	Bacteroidetes	Bacteroidia	Bacteroidales	Porphyromonadaceae		
2.420	1.759	0.428	0.498	577228	Bacteroidetes	Bacteroidia	Bacteroidales	S24-7		
0.186	0.022	0.050	0.187	New.ReferenceOTU2170	Bacteroidetes	Bacteroidia	Bacteroidales	BS11		
0.780	1.195	0.487	0.421	102513	Bacteroidetes	Bacteroidia	Bacteroidales	RF16		
1.024	0.341	0.310	0.187	16915	Bacteroidetes	Bacteroidia	Bacteroidales	p-2534-18B5		
0.000	0.021	0.010		1143674	Tenericutes	Mollicutes	Mycoplasmatales	Mycoplasmataceae		
0.366	0.002	0.178	0.350	1654477	Fusobacteria	Fusobacteriia	Fusobacteriales	Fusobacteriaceae		
0.146	0.279	0.063	0.790	New.ReferenceOTU3606	Tenericutes	Mollicutes	Anaeroplasmatales	Anaeroplasmataceae		
0.045	0.006	0.017	0.187	368490	Firmicutes	Bacilli	Turicibacterales	Turicibacteraceae		
0.027	0.073	0.023	0.814	349024	Firmicutes	Bacilli	Lactobacillales	Streptococcaceae		
1.600	2.247	0.447	0.923	302975	Firmicutes	Bacilli	Lactobacillales	Lactobacillaceae		
0.996	1.481	0.250	0.744	510572	Firmicutes	Clostridia	Clostridiales	Veillonellaceae		
0.915	0.888	0.245	0.807	215963	Firmicutes	Clostridia	Clostridiales	Clostridiaceae		
0.034	0.011	0.007	0.187	New.ReferenceOTU2707	Firmicutes	Clostridia	Clostridiales	Peptococcaceae		
0.021	0.004	0.007	0.199	712677	Firmicutes	Clostridia	Clostridiales	Peptostreptococcaceae		
0.196	0.176	0.044	0.814	302433	Firmicutes	Clostridia	Clostridiales	[Mogibacteriaceae]		
10.460	9.920	1.615	0.744	851865	Firmicutes	Clostridia	Clostridiales	Ruminococcaceae		
**Genus**										
4.770	4.377	0.990	0.986	New.CleanUp.ReferenceOTU122441	Firmicutes	Clostridia	Clostridiales	Lachnospiraceae	Roseburia	
0.603	0.284	0.126	0.380	1029949	Firmicutes	Clostridia	Clostridiales	Lachnospiraceae	Lachnospira	
0.022	0.011	0.008	0.789	4364564	Firmicutes	Clostridia	Clostridiales	Lachnospiraceae	Butyrivibrio	
0.104	0.334	0.070	0.332	1076587	Firmicutes	Clostridia	Clostridiales	Lachnospiraceae	Dorea	
0.286	0.233	0.077	0.747	696563	Firmicutes	Clostridia	Clostridiales	Lachnospiraceae	Blautia	
0.016	0.032	0.011	0.918	New.ReferenceOTU2736	Firmicutes	Clostridia	Clostridiales	Lachnospiraceae	Epulopiscium	
0.004	0.041	0.018	0.918	367139	Actinobacteria	Coriobacteriia	Coriobacteriales	Coriobacteriaceae	Slackia	
0.040	0.046	0.014	0.936	363794	Actinobacteria	Coriobacteriia	Coriobacteriales	Coriobacteriaceae	Collinsella	
0.148	0.154	0.058	0.906	New.ReferenceOTU3654	Fibrobacteres	Fibrobacteria	Fibrobacterales	Fibrobacteraceae	Fibrobacter	
4.362	2.778	0.993	0.378	924224	Spirochaetes	Spirochaetes	Spirochaetales	Spirochaetaceae	Treponema	
0.044	0.299	0.057	0.334	587570	Proteobacteria	Gammaproteobacteria	Aeromonadales	Succinivibrionaceae	Anaerobiospirillum	
0.693	0.654	0.305	0.986	517804	Proteobacteria	Gammaproteobacteria	Aeromonadales	Succinivibrionaceae	Succinivibrio	
0.000	0.002	0.000	0.360	9498	Proteobacteria	Gammaproteobacteria	Pasteurellales	Pasteurellaceae	Aggregatibacter	
0.098	0.428	0.135	0.211	359779	Proteobacteria	Gammaproteobacteria	Pasteurellales	Pasteurellaceae	Actinobacillus	
0.195	0.379	0.075	0.647	333380	Proteobacteria	Betaproteobacteria	Burkholderiales	Alcaligenaceae	Sutterella	
0.021	0.013	0.006	0.609	360508	Proteobacteria	Betaproteobacteria	Burkholderiales	Oxalobacteraceae	Oxalobacter	
0.969	0.585	0.327	0.582	25453	Deferribacteres	Deferribacteres	Deferribacterales	Deferribacteraceae	Mucispirillum	
0.364	0.410	0.097	0.918	4453773	Proteobacteria	Deltaproteobacteria	Desulfovibrionales	Desulfovibrionaceae	Desulfovibrio	
0.021	0.022	0.010	0.380	New.ReferenceOTU2103	Proteobacteria	Deltaproteobacteria	Desulfovibrionales	Desulfovibrionaceae	Bilophila	
7.330	10.015	1.639	0.918	113756	Proteobacteria	Epsilonproteobacteria	Campylobacterales	Campylobacteraceae	Campylobacter	
0.246	1.114	0.248	0.211	311173	Proteobacteria	Epsilonproteobacteria	Campylobacterales	Helicobacteraceae	Helicobacter	
3.097	0.443	1.338	0.986	New.ReferenceOTU2302	Bacteroidetes	Bacteroidia	Bacteroidales	Bacteroidaceae	Bacteroides	
44.876	45.018	3.496	0.893	568118	Bacteroidetes	Bacteroidia	Bacteroidales	Prevotellaceae	Prevotella	
0.493	0.282	0.121	0.770	4435235	Bacteroidetes	Bacteroidia	Bacteroidales	[Paraprevotellaceae]	YRC22	
13.588	15.662	2.129	0.647	20534	Bacteroidetes	Bacteroidia	Bacteroidales	[Paraprevotellaceae]	[Prevotella]	
3.232	2.798	0.496	0.687	300853	Bacteroidetes	Bacteroidia	Bacteroidales	[Paraprevotellaceae]	CF231	
0.000	0.003	0.001	0.378	4307122	Bacteroidetes	Bacteroidia	Bacteroidales	[Odoribacteraceae]	Odoribacter	
1.440	1.182	0.240	0.434	921813	Bacteroidetes	Bacteroidia	Bacteroidales	Porphyromonadaceae	Parabacteroides	
0.080	0.000	0.028	0.602	New.ReferenceOTU1695	Bacteroidetes	Bacteroidia	Bacteroidales	Porphyromonadaceae	Paludibacter	
0.000	0.029	0.014		1143674	Tenericutes	Mollicutes	Mycoplasmatales	Mycoplasmataceae	Mycoplasma	
0.503	0.002	0.244	0.380	1654477	Fusobacteria	Fusobacteriia	Fusobacteriales	Fusobacteriaceae	Fusobacterium	
0.102	0.231	0.088	0.211	New.ReferenceOTU3606	Tenericutes	Mollicutes	Anaeroplasmatales	Anaeroplasmataceae	Anaeroplasma	
0.084	0.008	0.034	0.211	368490	Firmicutes	Bacilli	Turicibacterales	Turicibacteraceae	Turicibacter	
0.035	0.095	0.029	0.906	349024	Firmicutes	Bacilli	Lactobacillales	Streptococcaceae	Streptococcus	
2.292	2.944	0.618	0.986	302975	Firmicutes	Bacilli	Lactobacillales	Lactobacillaceae	Lactobacillus	
0.000	0.007	0.002	0.211	25947	Firmicutes	Clostridia	Clostridiales	Veillonellaceae	Acidaminococcus	
0.045	0.057	0.010	0.986	916143	Firmicutes	Clostridia	Clostridiales	Veillonellaceae	Phascolarctobacterium	
0.040	0.056	0.021	0.918	149335	Firmicutes	Clostridia	Clostridiales	Veillonellaceae	Mitsuokella	
1.224	1.642	0.307	0.747	510572	Firmicutes	Clostridia	Clostridiales	Veillonellaceae	Anaerovibrio	
0.017	0.019	0.007	0.918	266210	Firmicutes	Clostridia	Clostridiales	Veillonellaceae	Megasphaera	
0.000	0.032	0.011	0.796	264552	Firmicutes	Clostridia	Clostridiales	Veillonellaceae	Dialister	
0.586	0.564	0.226	0.906	215963	Firmicutes	Clostridia	Clostridiales	Clostridiaceae	Clostridium	
0.055	0.015	0.014	0.211	New.ReferenceOTU2707	Firmicutes	Clostridia	Clostridiales	Peptococcaceae	rc4-4	
0.109	0.001	0.047	0.171	1112364	Firmicutes	Clostridia	Clostridiales	[Mogibacteriaceae]	Anaerovorax	
2.973	3.061	0.856	0.918	851865	Firmicutes	Clostridia	Clostridiales	Ruminococcaceae	Faecalibacterium	
1.263	0.600	0.278	0.211	148925	Firmicutes	Clostridia	Clostridiales	Ruminococcaceae	Ruminococcus	
2.535	1.972	0.394	0.211	310886	Firmicutes	Clostridia	Clostridiales	Ruminococcaceae	Oscillospira	
0.012	0.036	0.011	0.986	1111191	Firmicutes	Clostridia	Clostridiales	Lachnospiraceae	[Ruminococcus]	
0.961	1.017	0.230	0.796	369027	Firmicutes	Clostridia	Clostridiales	Lachnospiraceae	Coprococcus	
0.022	0.012	0.003	0.796	New.ReferenceOTU1761	Firmicutes	Clostridia	Clostridiales	Lachnospiraceae	Anaerostipes	
**Species**										
0.070	0.014	0.022	0.687	362501	Firmicutes	Clostridia	Clostridiales	Lachnospiraceae	Coprococcus	eutactus
0.074	0.239	0.093	0.378	1076587	Firmicutes	Clostridia	Clostridiales	Lachnospiraceae	Dorea	formicigenerans
0.111	0.142	0.038	0.434	363794	Actinobacteria	Coriobacteriia	Coriobacteriales	Coriobacteriaceae	Collinsella	aerofaciens
0.057	0.064	0.036	0.918	638485	Fibrobacteres	Fibrobacteria	Fibrobacterales	Fibrobacteraceae	Fibrobacter	succinogenes
0.101	0.051	0.027	0.211	360508	Proteobacteria	Betaproteobacteria	Burkholderiales	Oxalobacteraceae	Oxalobacter	formigenes
2.286	1.676	0.803	0.986	25453	Deferribacteres	Deferribacteres	Deferribacterales	Deferribacteraceae	Mucispirillum	schaedleri
0.176	0.014	0.050	0.918	365496	Bacteroidetes	Bacteroidia	Bacteroidales	Bacteroidaceae	Bacteroides	plebeius
15.559	18.297	2.129	0.747	591785	Bacteroidetes	Bacteroidia	Bacteroidales	Prevotellaceae	Prevotella	stercorea
71.005	68.629	3.407	0.918	568118	Bacteroidetes	Bacteroidia	Bacteroidales	Prevotellaceae	Prevotella	copri
0.031	0.263	0.071	0.796	349024	Firmicutes	Bacilli	Lactobacillales	Streptococcaceae	Streptococcus	luteciae
0.005	0.051	0.016	0.918	New.ReferenceOTU3048	Firmicutes	Bacilli	Lactobacillales	Lactobacillaceae	Lactobacillus	mucosae
0.086	0.104	0.039	0.211	149335	Firmicutes	Clostridia	Clostridiales	Veillonellaceae	Mitsuokella	multacida
9.848	9.905	2.879	0.918	851865	Firmicutes	Clostridia	Clostridiales	Ruminococcaceae	Faecalibacterium	prausnitzii
0.368	0.262	0.111	0.893	369602	Firmicutes	Clostridia	Clostridiales	Ruminococcaceae	Ruminococcus	bromii
0.117	0.008	0.057	0.770	New.ReferenceOTU3836	Firmicutes	Clostridia	Clostridiales	Ruminococcaceae	Ruminococcus	flavefaciens
0.047	0.241	0.094	0.647	1111191	Firmicutes	Clostridia	Clostridiales	Lachnospiraceae	[Ruminococcus]	gnavus
0.058	0.040	0.012	0.687	New.CleanUp.ReferenceOTU17239	Firmicutes	Clostridia	Clostridiales	Lachnospiraceae	Roseburia	faecis
